# Scorpion envenomation-associated myocarditis: A systematic review

**DOI:** 10.1371/journal.pntd.0011219

**Published:** 2023-04-05

**Authors:** Reza Fereidooni, Saeedreza Shirzadi, Seyyed Hamidreza Ayatizadeh, Mabrouk Bahloul, Amirali Tavangar, Seyed Alireza Zomorodian, Amirhossein Roshanshad, Ali Ardekani

**Affiliations:** 1 Health Policy Research Center, Institute of Health, Shiraz University of Medical Sciences, Shiraz, Iran; 2 Student Research Committee, Shiraz University of Medical Sciences, Shiraz, Iran; 3 Department of Intensive Care, Habib Bourguiba University Hospital. Faculté de medicine de Sfax. Sfax University. Sfax, Tunisia; 4 Digestive Health Institute, University of California, Irvine Medical Center, Orange, California, United States of America; 5 Shiraz Nephro-Urology Research Center, Shiraz University of Medical Sciences, Shiraz, Iran; 6 School of Medicine, Shiraz University of Medical Sciences, Shiraz, Iran; Muséum National d’Histoire Naturelle, FRANCE

## Abstract

**Background:**

Scorpion envenomation is associated with several complications. One of the most serious complications is the cardiac involvement in the form of myocarditis that remains the main reason for mortalities associated with scorpion envenomation. The present review aims to elucidate clinical and paraclinical findings associated with scorpion-related myocarditis, and to explore different management strategies and subsequent outcomes.

**Methods:**

We searched PubMed, Web of Science, Scopus, and Google Scholar for articles related to keywords of myocarditis associated with scorpion envenomation up to May 1, 2022. Each article was carefully reviewed by two independent researchers. In case of disagreement for inclusion, we sought a third researcher opinion.

**Results:**

A total of 703 cases from 30 case reports and 34 case series were included in our review. Myocarditis associated with scorpion envenomation was usually reported in children presenting with cardiopulmonary symptoms including pulmonary edema (60.7%) and shock or hypotension (45.8%). The most common ECG findings are sinus tachycardia (82%) followed by ST-T changes (64.6%). The management typically included inotropes (especially dobutamine), prazosin, diuretics, nitroglycerine and digoxin, when indicated. Mechanical ventilation was required in 36.7% of the patients. Mortality in confirmed scorpion-related myocarditis cases is estimated at 7.3%. Almost all survived cases showed rapid recovery and improvement in the left ventricular function.

**Conclusion:**

Even though myocarditis associated with scorpion envenomation is rare, it remains a serious and in some of cases a fatal consequence of scorpion sting. In case of relative presentations, particularly in envenomed children, diagnosis of myocarditis should be considered. Early screening using serial cardiac markers and echocardiography can guide the treatment. Prompt treatment that focuses on cardiogenic shock and pulmonary edema usually results in a favorable outcome.

## Author summary

Scorpion sting can cause a variety of complications. One of the most dangerous complications associated with some scorpion venoms is damage to the heart in the form of myocarditis. Myocarditis is the inflammation of the heart muscle. The mechanism of myocarditis in scorpion sting is unique. The scorpion venom triggers a massive release of epinephrine and norepinephrine hormones, which affect the heart. At first, rapid heartbeats, hypertension, and agitation occurs. Then, as the result of the cardiac dysfunction, respiratory distress, fluid buildup in the lungs and a drop in blood pressure will develop. Children are particularly vulnerable to this condition. Electrocardiograms will reveal signs of heart strain and even dangerous arrhythmias. Serial heart enzyme measurements can help diagnose this condition. Echocardiograms show heart function decline. Respiratory support is critical. Prazosin is helpful for hypertension. Heart failure and its complications are the main targets of treatment. Dobutamine has shown the most promise in treating heart failure. We estimate the mortality rate at 7.4% in the reported cases. With timely management this condition is usually benign, recovery is quick, and survivors rarely experience long-term complications. Physicians should be aware of this potential complication, screen for it and begin appropriate treatment as soon as possible.

## Introduction

Scorpion envenomation represents a multifaceted clinical entity and a medical emergency with widespread incidence, particularly in tropical and subtropical areas [1]. The high frequency and potentially severe complications make scorpion envenomation a considerable burden on public health and safety in these regions [1–3]. Additionally, a growing concern is being mounted as factors like climate change are projected to alter the spatiotemporal distribution of clinically significant scorpion populations and human-scorpion interactions [4–6]. The incidence and mortality of scorpion envenomation have reportedly increased in the last decade [7–9].

Clinical manifestations of scorpion envenomation can be separated into local and systemic clusters, with local manifestations including pain ranging from minimal to severe, pruritus, erythema, swelling, inflammation, ecchymosis, blisters, necrosis and gangrene, and systemic manifestations including tachycardia, tachypnea, shock, respiratory distress, neurologic impairments like agitation and altered mental status, nausea, vomiting, and hematuria [10–17]. A consensus classification of the severity of these clinical manifestations comprises four classes, with class I being only local, class II minor systemic, class III major systemic, and class IV lethal manifestations [15]. The severity of manifestations is affected by scorpion variables of species, size, type of toxin, state of telson venom duct, number of stings, time to treatment administration, the venom quantity injected, and patient variables of anatomical location of sting, age, weight, and health status [1,18].

One of the most important systemic involvements of scorpion envenomation is cardiac presentation [19]. The nature of cardiac dysfunction in scorpion envenomation appears to be multifactorial [19]. It has been labeled both myocarditis and cardiomyopathy in the literature [19,20]. Pulmonary edema and cardiogenic shock, the most dreaded complications of scorpion envenomation, are associated with cardiac involvement [16,19,21,22]. This condition is highly correlated with mortality and poor outcomes, with children younger than 5 years being the most vulnerable [2].

The mechanism by which scorpion envenomation causes myocarditis has been mainly associated with the release of catecholamines and vasoactive peptides [22]. Scorpion venom’s neurotoxins affect voltage-gated sodium (Na) and potassium (K) channels leading to neuronal excitation and release of a large quantity of acetylcholine, epinephrine, and norepinephrine from the sympathetic and parasympathetic nerve endings and adrenal medulla [1,3,22–25]. Vasoconstriction caused by the catecholamines results in an afterload increase [19]. Furthermore, myocardial ischemia is shown in scorpion envenomation, which may be another contributing factor [26,27]. Ischemia may be caused by the vasoconstriction and increased myocardial contractility and oxygen demand, induced by catecholamine and sympathetic storm [26,28]. Finally, a direct effect of venom on the myocardium by disrupting the ion concentrations in myocytes has been suggested [29,30]. All of these contribute to manifestations of myocardial stunning or stress-induced cardiomyopathy [22].

With increasing incidence of scorpion envenomation, changing population dynamics, and climate change, the need to better understand it to mitigate its growing incidence and effects has become more pivotal [1,5]. The mortality associated with scorpion envenomation is mainly due to myocarditis and acute pulmonary edema [31]. Also, the occurrence of scorpion envenomation is concentrated in developing regions with less access to optimal medical and critical care resources. Hence, it necessitates better evidence and understanding to recognize severe cases earlier and allocate these limited resources to improve outcomes properly. The present systematic review aims to elucidate clinical, electrocardiographic, and echocardiographic findings associated with scorpion-related myocarditis, as well as to explore different management strategies and subsequent outcomes and to establish the associated mortality rate.

## Material and methods

To report the present study, we followed the standard protocol of Preferred Reporting Items for Systematic reviews and Meta-Analyses (PRISMA) [32]. The PRISMA checklist is available in the S1 Table.

### Search strategy

At the end of April 2022, a preliminary search was done to retrieve the best keywords. On May 1, the primary search was done in PubMed, Scopus, and Web of Science utilizing the relevant keywords and MeSH terms representing “scorpion”, “myocarditis”, and “cardiomyopathy” (S2 Table). We searched the first 100 results of Google Scholar to gather grey literature as well. A forward and backward reference checking was performed for the final included studies. All citations were imported to Endnote, and duplicates were removed. Given that the terms “cardiomyopathy”, “myocardial damage”, and “myocarditis” are used in the literature to refer to the same entity in scorpion envenomation, they are used interchangeably in this study.

### Screening

Two independent authors screened the titles and abstracts of the studies according to the inclusion and exclusion criteria. Then, the full-texts of the remainder of the studies were assessed by two independent authors. A third author was consulted in cases of dissimilarities in the screening process. The case definition for inclusion was based on studies with confirmed scorpion stings and one of the following:

- Fulfilling histological or immunohistological criteria for myocarditis or inflammatory cardiomyopathy [33]
- Fulfilling two of the following criteria: (i) an elevated level of troponin T or troponin I, (ii) imaging or hemodynamic proof of acute impaired cardiac function using echocardiography, cardiac MRI, nuclear imaging, or pulmonary artery catheterization, (iii) clinical symptoms of cardiovascular dysfunction (e.g., dyspnea, chest pain, pulmonary edema or shock)

As exclusion criteria, we factored in the case series in which patients with myocardial damage were grouped with other cases; hence, the case number and other data could not be attributed to the cases with myocarditis unambiguously. In addition, we excluded those studies where no definition was presented for diagnosing myocarditis or the cause of cardiac dysfunction was proven to be myocardial infarction or other causes. Also, all types of review articles and animal studies were excluded. No exclusions were made due to the date, language, or country of publication.

### Data extraction

Two independent researchers extracted the data. The first author’s name, year of publication, study design, description of the case or method of the study, ECG and echocardiography findings, treatment strategies, and outcome of the patients were extracted when available. The prevalence of the aforementioned findings is reported in the tables whenever they could be attributed specifically to myocarditis cases. Similarly, to conduct descriptive analysis across all cases, only the publications that reported the presence and frequency of the findings of interest were taken into account. To calculate mortality, we excluded the papers in which only autopsy cases were presented and those in which myocarditis was not the leading cause of death. In case of disagreements, consultation with the most expert co-authors was put forth until a consensus was reached.

## Results

**Fig 1:**
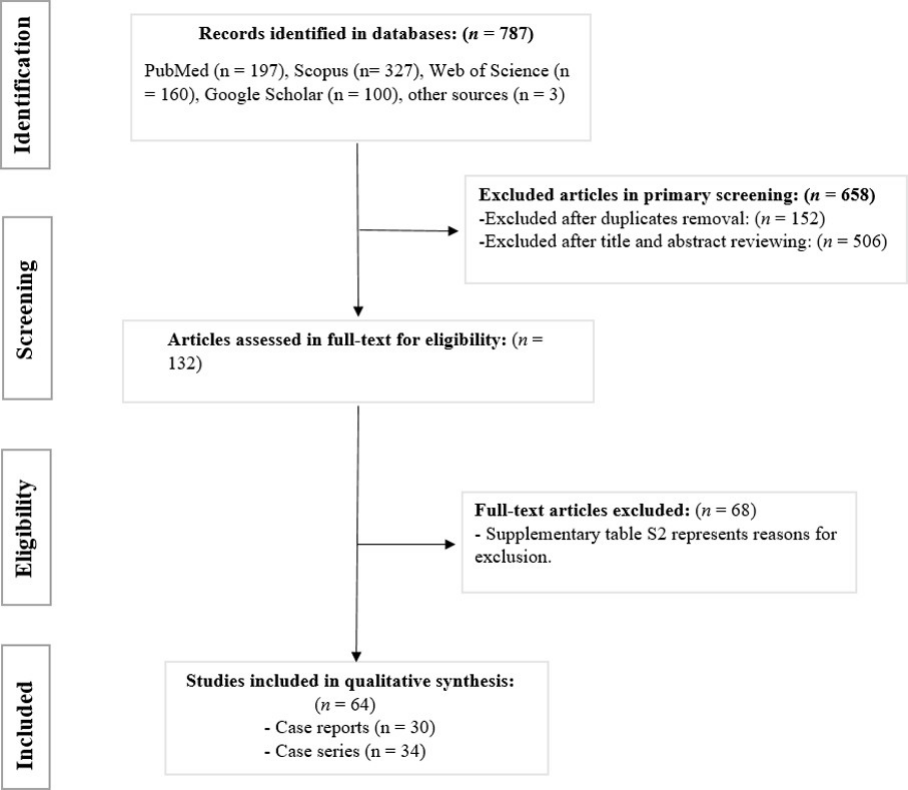
The flow-diagram of the study.

Searching through the databases, 787 citations were found. After removing the duplicates, the titles and abstracts of 509 studies were screened. Then the full texts of 132 studies were assessed, and 64 were included in the final review (Fig 1). S3 Table provides the excluded studies along with the reasons for exclusion. A total of 703 cases, consisting of 669 from 34 case series [13,14,18,26,27,34–62] and 34 from 30 case report articles [63–92], were extracted from the literature.

### General characteristics of the studies

**Fig 2:**
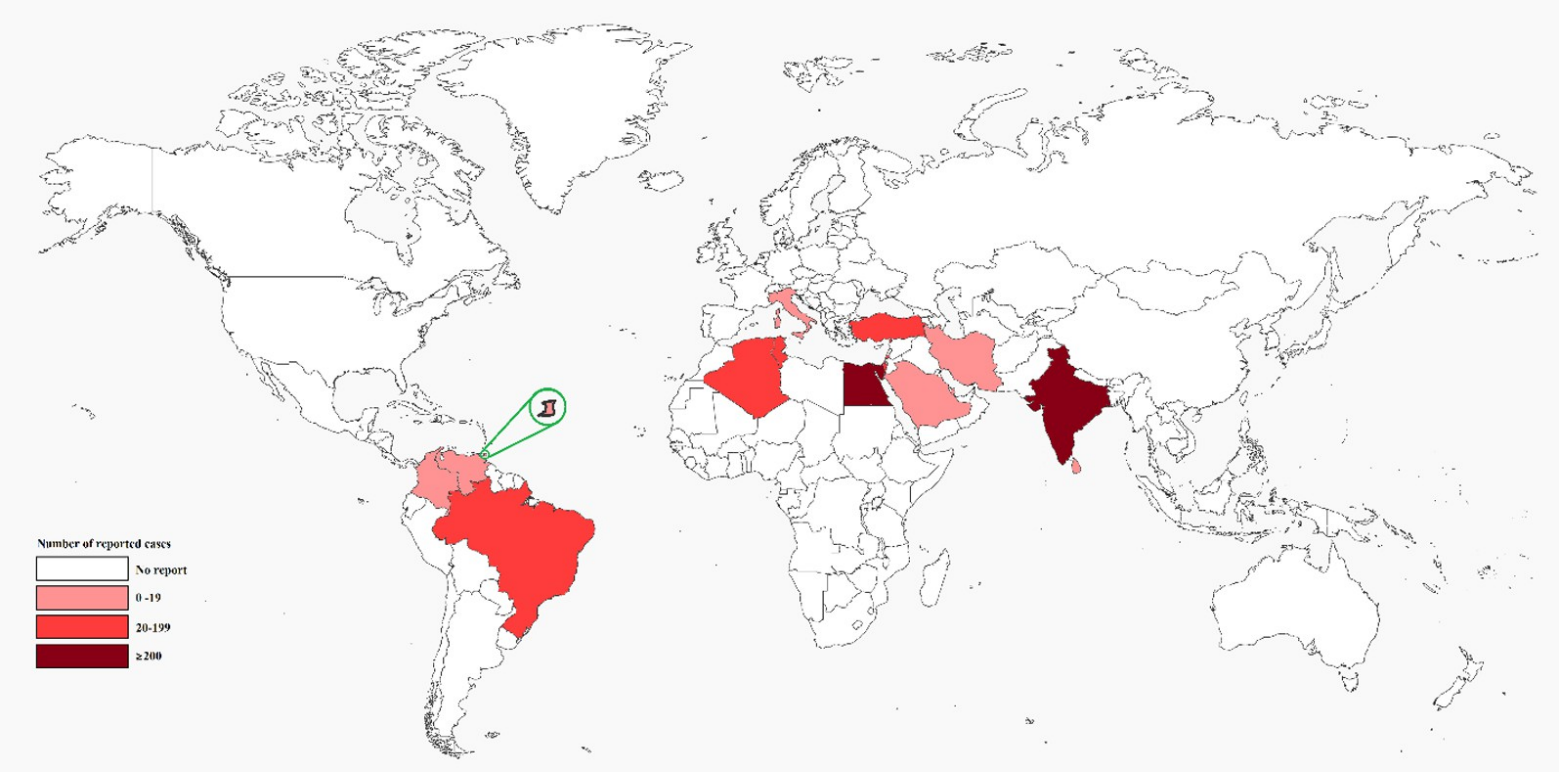
Geographic distribution of cases.

Regarding geographical distribution, cases were distributed across 15 countries with either arid or tropical climates. The largest number of articles (n = 15) were from India and most cases (n = 256) were from Egypt. The geographical distribution of the reported cases can be seen in Fig 2. The most commonly reported species were Tityus serrulatus in Brazil, Hottentotta tamulus (previously known as Mesobuthus tamulus) in India, and Leiurus quinquestriatus and Androctonus australis in the Middle East and North Africa region, all of which are from the family Buthidae. The majority of the cases were children with 92% (564 of 613) being under 18 years of age and 30.2% (52 of 172) being under 5.

An overview of the included case series papers is shown in Tables 1 and 2 and case reports in S4 Table. In these tables, only the information regarding the cases which fulfilled our criteria is presented.

**Table 1:** Description of included case series*.

Authors	Country	Year	Myocarditis case number	Scorpion species	Age of myocarditis cases	Cardiac biomarker/Echocardiography Other diagnostic methods
**Abdel Baseer et al. [34]**	Egypt	2021	28	-	12 (42.8%) under 5 16 (57.2%) above 5	+/+
**Abroug et al. [35]**	Tunisia	1991	5	Androctonus australis, hector or garzonii,	21.6 (range 12 to 34)	-/- Pulmonary artery catheterization
**Abroug et al. [36]**	Tunisia	1995	9	Androctonus australis	22 (range 15 to 34)	-/+
**Amaral et al. [37]**	Brazil	1991	5	Tityus serrulatus	5.6 (range 3 to 9)	+/+
**Amaral et al. [38]**	Brazil	1993	3 (+3 that were repeated from the above article)	Tityus serrulatus	4.66 (range 2 to 9)	-/+
**Bahloul et al. [39]**	Tunisia	2002	6	-	-	-/+ Pulmonary artery catheterization
**Bahloul et al. [26]**	Tunisia	2004	6	Androctonus australis, hector or garzonii	15.83 (range 4 to 29)	-/+ Scintigraphy
**Bucaretchi et al. [40]**	Brazil	1995	6	Tityus serrulatus, Tityus bahiensis	3.8 (range 1 to 8)	+/+
**Çağlar et al. [41]**	Turkey	2015	9	-	-	+/+
**Çelik et al. [42]**	Turkey	2021	17	-	-	+/+
**Chaari et al. [43]**	Tunisia	2014	3	Androctonus australis	Range 18 to 23	-/- Transpulmonary thermodilution
**Cupo, et al. [44]**	Brazil	1994	4	Tityus serrulatus	Range 4 to 6	+/+ Pathologic confirmation
**Cupo and Hering [45]**	Brazil	2002	8	Tityus serrulatus	5.8 (range 2 to 9)	+/+
**Cupo et al. [46]**	Brazil	2007	10	Tityus serrulatus	6.9 (range 2 to 12)	+/+ Scintigraphy
**Daisley et al. [47]**	Trinidad and Tobago	1999	4	Tityus trinitatis	Range 3 to 5	-/- Pathologic confirmation
**Delma [48]**	Algeria	2012	20	-	Median 17.5 (range 3 to 50)	-/+
**Figueirdo et al. [27]**	Brazil	2009	10	-	-	+/- Scintigraphy
**Gökay et al. [49]**	Turkey	2018	7	-	Median of 6 years and 3 months (IQR 12–132 months)	+/+
**Gueron and Yaron [50]**	Israel	1970	7	Leiurus quinquestriatus (old name: Buthus quinquestriatus)	3.4 (range 8 months to 9 years old)	-/- Pathologic confirmation
**Gueron et al. [51]**	Israel	1990	3	Leiurus quinquestriatus	6.3 (range 3 to 14)	+/+
**Hering et al. [62]**	Brazil	1993	12	Tityus serrulatus	8.4 (range 4 to 18)	
**Khalaf et al. [52]**	Egypt	2021	211	-	Range 2 to 17	+/+
**Kumar et al. [53]**	Saudi Arabia	1992	12	-	-	-/+
**Kumar et al. [18]**	India	2015	24	Hottentotta tamulus (old name: Mesobuthus tamulus)	< 13 years	+/+
**Kumar and Prasad [13]**	India	2015	21	-	-	-/+
**Mazzei de Dàvila et al. [54]**	Venezuela	2002	7	Tityus zulianus	-	-/+
**Meki et al. [55]**	Egypt	2002	17	Buthus occitanus or Leiurus quinquestriatus	-	+/+
**Nouira et al. [56]**	Tunisia	1995	8	Androctonus australis	28±16 (range 14 to 68)	-/- Pulmonary artery catheterization
**Prakash et al. [57]**	India	2021	21	-	-	+/+
**Prasad et al. [14]**	India	2011	38	-	-	-/+
**Prasad et al. [58]**	India	2020	25	-	-	+/+
**Rajasekhar and Mohan [59]**	India	2004	24	Hottentotta tamulus (old name: Mesobuthus tamulus)	23.2±11.7	+/+
**Sagarad et al. [60]**	India	2012	60	-	Majority were below 12 years	+/+
**Sofer et al. [61]**	Israel	2013	19	Leiurus quinquestriatus	-	+/+

Abbreviations: (-): not mentioned or not attributable

*The case number and age in this table only apply to cases with myocardial dysfunction; they do not apply to all of the study participants.

**Table 2:** Clinical findings, treatment, and outcome in case series*.

Authors	Clinical presentation of myocarditis cases	Echocardiography findings of myocarditis	ECG findings	Treatment	Mortality
**Abdel Baseer et al. [34]**	Respiratory distress (67.8%), pulmonary edema (39.2%), hypotension (46.4%), gallop rhythm (42.8%), fever (60.7%), priapism (3.5%), abdominal pain (21.4%), convulsion (7.1%), bradycardia (9.5%), hypertension (7.1%), wheeze (28.6%)	LV dysfunction (100%), in the form of LV dilatation, decreased FS (21.37±4.59) and LVEF (40.25±6.75), transient functional mitral incompetence as a sign of left ventricular dilatation (17.9%), global hypokinesia (42.9%)	Sinus tachycardia (75%), ST-segment changes (17.9%), sinus bradycardia (7.1%), low voltage complexes (17.9%), AV block (10.7%), ventricular ectopics (7.1%), T-wave inversion (14.3%)	Mechanical ventilation (46.4%), inotropes, prazosin	3
**Abroug et al. [35]**	Pulmonary edema with bilateral and diffuse confluent opacities on CXR (100%), severe hypoxemia with peripheral signs of hypoperfusion (100%), tachycardia with normal or moderately depressed blood pressure (100%)	-	Premature ventricular beats (20%), tachycardia (100%)	Mechanical ventilation (80%), dobutamine (60%)	2
**Abroug et al. [36]**	Pulmonary edema (100%), peripheral circulatory failure (77.8%)	Hypokinetic LV (100%), markedly depressed LV systolic function (FS = 12±6%; EF = 26±12%) with associated diastolic dysfunction, transient mitral regurgitation (55.5%)	ST segment depression (55.6%), tachycardia (77.8%)	SAV, hydrocortisone, mechanical ventilation (44.5%), dobutamine	1
**Amaral et al. [37]**	Vomiting (100%), hyperthermia (40%), restlessness (60%), tachycardia (100%), tachypnea (100%), and Pulmonary edema (60%)	Depressed LVEF (100%) characterized by poor motion of the interventricular septum or decreased motion of the left ventricular posterior wall and decreased left ventricular fractional shortening, or a combination of these, mitral regurgitation (60%)	Sinus tachycardia (100%). acute myocardial infarction-like pattern (60%), frequent ventricular premature beats with periods of bigeminy (20%)	SAV, dipyrone, metoclopramide, oxygen, diuretics, and digitalis.	0
**Amaral et al. [38]**	Leukocytosis (100%), Pulmonary edema (100%), shock (66%).	Hypokinesia of the interventricular septum and LV posterior wall, and decreased LVFS (33%).	Sinus tachycardia (100%), myocardial ischemia pattern (33%)	SAV, dipyrone, metoclopramide, oxygen, furosemide, deslanoside, mechanical ventilation (66%), dobutamine or dopamine.	2
**Bahloul et al. [39]**	Pulmonary edema (100%), tachycardia, tachypnea,	LVEF < 40% (in 1 patient)	-	Mechanical ventilation, inotropes	-
**Bahloul et al. [26]**	Fever (83.3%), shock (33.3%), pulmonary edema (100%)	Decreased LVEF (100%), mitral regurgitation (33.3%), interventricular septum hypokinesia or hypokinesia (100%), posterior left wall hypokinesia (50%)	Tachycardia (100%), ST change (33.3%), T inversion (50%), tall T wave (33.3%), branch block (16.7%)	Mechanical ventilation (33.3%), dobutamine	0
**Bucaretchi et al. [40]**	Pulmonary edema (83.3%), shock (66.6%)	Depressed LVEF (100%)	MI-pattern (100%), sinus tachycardia, prominent T and U waves, ventricular premature beats	SAV, dobutamine, mechanical ventilation (100%), digoxin (83.3%), furosemide	1
**Çelik et al. [42]**	Pulmonary edema (0%)	Altered cardiac function	-	SAV, tetanus toxoid, doxazosin	0
**Çağlar et al. [41]**	Pulmonary edema (100%)	Reduced LVEF and FS	Tachycardia	SAV, dobutamine, mechanical ventilation	2
**Chaari et al. [43]**	Pulmonary edema (100%), hypotension (100%)	-	Tachycardia (100%)	SAV, mechanical ventilation (100%), dobutamine	0
**Cupo, et al. [44]**	Vomiting (100%), hypotension (75%), hypothermia (25%), pulmonary edema (100%), tachy-dyspnea (100%), tachycardia (100%), cold extremities (100%), hyperglycemia (25%), leukocytosis (25%), sweating (50%), sialorrhea (25%), abdominal pain (25%), change in level of consciousness (100%)	-	Tachycardia (100%), ST segment depression (25%)	SAV, oxygen, dopamine, digitalis, diuretics, mechanical ventilation (25%)	3
**Cupo and Hering [45]**	Local pain (100%), sweating (100%), sialorrhea (87.5%), agitation/tremor (100%), hypothermia (100%), tachydyspnea (100%), pulmonary edema (62.5%), tachycardia (87.5%), hypertension (87.5%)	LV dysfunction with hypo- or dyskinesia (100%), dilated LV (85.7%), mitral or tricuspid regurgitation (85.7%)	Ventricular extrasystoles, sinus tachycardia and bradycardia, ST-T change	SAV, dobutamine, mechanical ventilation (16.7%)	0
**Cupo et al. [46]**	Pulmonary edema (70%), tachycardia (50%)	Mean LVEF = 30.6% (range 20%-44%)	Sinus tachycardia, ST-T change, Q waves	SAV, dobutamine	0
**Daisley et al. [47]**	Vomiting (100%), hypotension (75%), pulmonary edema (100%), tachy-dyspnea (100%), tachycardia (75%), cold extremities (50%), sialorrhea (25%), abdominal pain (25%), restlessness (100%)	-	Tachycardia (75%), ST change (50%)	Mechanical ventilation (50%), digoxin, diuretic, antiemetic, antibiotics, antihistamines, hydrocortisone and isotonic fluids	2
**Delma [48]**	Pulmonary edema (100%)	LVEF below the standard (100%), mitral regurgitation (50%), disorders of myocardiac kinetics, resembling myocardial akinesia or hypokinesia, mostly septal or anterior, global hypokinesia (25%), low FS (65%), telediastolic and telesystolic diameters (TDD and TSD, respectively) were within the normal range	-	Dobutamine	-
**Figueirdo et al. [27]**	Acute pulmonary edema (70%)	Decreased global LV systolic performance, significant abnormalities of the segmental wall motion	Sinus tachycardia, T-wave and ST changes (elevation and/or depression). transient “electrically inactive zone”	Vasoactive amines (60%)	0
**Gökay et al. [49]**	Vomiting (100%)	LVEF = 51.6±13.6 LVFS = 26.2±7.9	-	SAV, doxazosin, dobutamine	0
**Gueron et al. [50]**	Pulmonary edema (66%), shock (33%)	-	Myocardial infarction pattern, ST-T change	-	9
**Gueron et al. [51]**	Pulmonary edema (66%), hypertension (66%), hypotension (33%)	Poorly contracting wall motion (global) with decreased LVEF and normal wall thickness (100%), mitral incompetence (66%).	-	Symptomatic management, sublingual nifedipine	0
**Hering et al. [62]**	Tachydyspnea (100%), hypertension (66%), sialorrhea (91.7%), lacrimation (50%), abdominal pain (41.7%), nausea/vomiting (100%), agitation (100%), tremor (50%), chest pain (8.3%), priapism (14.3%)	Moderate to severe LV systolic dysfunction with decreased LVEF and FS (100%), LV dilation (41.7%), diffuse or segmental (especially interventricular septum) LV hypokinesia	Sinus tachycardia (100%), ventricular premature beats (66.7%), first degree atrioventricular block (41.7%), pathological Q waves (58.3%), ST depression/elevation (83.3%), T wave change (100%), prolonged QTc (66.7%), U wave (50%)	SAV, dopamine, dobutamine (8.3%), assisted ventilation (8.3%), bed rest, oxygen therapy via a nasal cannula, diuretic	0
**Khalaf et al. [52]**	-	Wall motion abnormalities and decreased EF	ST elevation	-	-
**Kumar et al. [53]**	Sinus tachycardia, gallop rhythm, peripheral vasoconstriction, fever, and tachypnea.	Compromised LV function; FS being less than 27% and LVEF less than 0.55. LV systolic dimension was 3.52±0.42 at diastole and 2.74±0.33 at systole, and marked decrease in contractility of the interventricular septum. The septum appeared flat on the initial M-mode recording, with virtually no systolic thickening.	-	-	1
**Kumar et al. [18]**	Hypotension (33%), encephalopathy (4%)	LV dysfunction	-	SAV, prazosin, oxygen, dobutamine mechanical ventilation (25%), sodium nitroprusside, intravenous nitroglycerine	1
**Kumar and Prasad [13]**	Tachypnea and cardiomegaly, muffled heart sounds and gallop rhythm (100%), acute pulmonary edema (52.4%).	LV dysfunction (100%) in the form of LV dilatation and all showed decreased LVEF below 20%, regional wall motion abnormalities in 7 (36.8%) cases ranging from akinesia to global hypokinesia of the LV, transient mitral incompetence in 3 (16% of cases with myocarditis)	Tachycardia, sinus bradycardia, ST-segment depression, tall T-waves, T-wave inversion, low voltage complexes	Dobutamine, prazosin (100%), IV fluid, diazepam, oxygen furosemide, mechanical ventilation	2
**Mazzei de Dàvila et al. [54]**	Pulmonary edema (100%) of varying severity	LVEF = 31±9, hypokinetic interventricular septum (83.3%), lateral hypokinesia (33%)	Sinus tachycardia, premature ventricular contractions, T wave inversion, ST elevation	Mechanical ventilation, angiotensin converting enzyme inhibitors, beta-adrenergic agonists, digitalis, diuretics	0
**Meki et al. [55]**	Pulmonary edema (52.9%), shock (58.8), gallop rhythm (88.2%), apical murmur (47%)	LVEF = 33.70±2.28 LVFS = 29.80±0.66	Heart block (47%), low voltage QRS (88.2%), ventricular ectopics (29.2%)	SAV (100%), vasopressors (58.8%), mechanical ventilation	5
**Nouira et al. [56]**	Tachycardia (75%), pulmonary edema (100%), peripheral circulatory failure (50%)	-	-	Dobutamine, nitrates, mechanical ventilation (37.5%), SAV (75%), hydrocortisone (87.5%)	1
**Prakash et al. [57]**	Vomiting, sweating, hypersalivation, cold extremities, hypotension, hypertension, priapism, tachycardia, bradycardia, chest x-ray change (33.3%)	Impairment in cardiac function (100%), hypokinesia of LV, decreased LVEF, pulmonary artery hypertension, grade I tricuspid regurgitation	ECG change (19.04%)	Inotropes (47.6%), milrinone (14.3%), mechanical ventilation (19%)	1
**Prasad et al. [14]**	Excessive perspiration (100%), cold extremities (100%), pulmonary edema (78.9%)	Decreased LVEF (100%)	-	Oxygen, dobutamine, prazosin, antihistamines, dexamethasone, mechanical ventilation (42.1%)	8
**Prasad et al. [58]**	Tachypnea, hypotension, S3 gallop, tachycardia, and pulmonary edema	LVEF = 41–50%: 10 patients LVEF = 31–40%: 7 patients LVEF < 30%: 1 patient	Sinus tachycardia, low voltage complex, ST segment elevation or depression, tall T wave or T wave inversion	Oxygen, intravenous fluid, inotropic i.e. dobutamine, prazosin, mechanical ventilation, and nitroglycerine in case of ARDS.	1
**Rajasekhar and Mohan [59]**	Breathlessness (63%), anxiety (100%), hypotension (79%), pulmonary edema (54%)	Varying degrees of LV dysfunction, diameter (mm) in diastole 47.6±6.2, diameter (mm) in systole 42.0±7.1, end-diastolic volume (ml): 108.7±31.9, end-systolic volume (ml): 81.3±30.9, Stroke volume (ml) 27.8±13.2, ejection fraction (%): 25.5±12.8. None had regional wall motion abnormalities, global hypokinesia, or dilated cardiomyopathy	Sinus tachycardia (100%), widening of QRS complex (21%)	L-carnitine, inotropic support, diuretics	0
**Sagarad et al. [60]**	Pulmonary edema (16.7%), hypotension (30%), tachycardia (62%), hypertension (33%), muffled heart sound/S3 gallop (52%)	Decreased LVEF (< 50%) in all myocarditis cases. LVEF of less than 40% in 20 patients (33.3%). RV dysfunction in 30 (50%) patients.	-	Mechanical ventilation, inotropic support (55%), prazosin	2
**Sofer et al. [61]**	Fever, decreased level of consciousness (56%), restlessness (95%), vomiting (100%), tachycardia (45%). shivering (72%), mottled skin (75%), dyspnea (32%)	Hypokinesia (100%), low FS (94.7%), and low EF (78.9%). One child (5.3%) had hypokinesia with borderline parameters. Mean arrival LVEF = 44.6±9	ST change (36.8%)	SAV, sublingual nifedipine or hydralazine (31.6%), oxygen (68.4%), mechanical ventilation (47.4%), dobutamine (84.2%), dopamine (21%), bolus fluid (74%)	0

Abbreviations: SAV: scorpion antivenom, LV: left ventricle, EF: ejection fraction, FS: fractional shortening, ARDS: acute respiratory distress syndrome, (-): not mentioned or not attributable

*The reported prevalence numbers and echocardiography findings in this table are reported if they were attributable only to cases with myocardial dysfunction; they do not apply to all of the study participants.

### Clinical and laboratory characteristics

Aside from the local signs and symptoms of the sting (i.e., pain and swelling of the sting site), which was present in almost all cases, restlessness, fever, sweating, vomiting, and priapism were the other more common manifestations that were reported regardless of the presence of myocarditis. Symptoms and clinical findings associated with myocarditis included respiratory distress (tachypnea and/or dyspnea), mostly associated with pulmonary edema, cold or cyanotic extremities, hypotension or shock, increased heart rate, and gallop rhythm on heart auscultation. Among these, respiratory distress [26,28,34,36–38,40,43–48,51,59,63–71,73–92] and increased heart rate (tachycardia) [13,27,34,36–39,43,44,47,53,56,58,59,61,64–66,68,69,74,75,77–89] were the most common manifestations affecting 94.8% (145 from 153) and 82% (137 from 167) of the patients, respectively. In studies where the prevalence of pulmonary edema could be determined unequivocally, it was observed in 60.7% (246 from 405) of patients [13,27,34,36–39,42–44,47,48,51,56,58,59,64–66,69,75,78–89]. Shock or hypotension was seen in 45.8% (108 from 236) of patients [14,18,26,27,34,36–38,40,43–47,49–51,55,56,60,61,63–92]. Hypertension, despite cardiac dysfunction, was another notable presentation reported in some cases [34,50,51,57,60,66,74,76,82,84]. Sinus tachycardia was the most common ECG reading [13,27,34–38,43,44,47,53,58–62,64–66,68,69,74,75,77–89], followed by ST-T changes [13,27,34,36–38,44,47,58,61,62,64,66,68,69,72,75,77–80,82,83,85,89]. ST-T changes, including ST segment elevation or depression, T inversion, or tall T waves, were present in 64.6% (84 from 130). Ventricular tachycardia was another important finding [71,75,76,84]. Laboratory findings associated with myocarditis were raised levels of cardiac troponin T [49,57,61,64,70,72,75,87], troponin I [34,41,42,45,46,49,58,60,65,66,74,76,78,79,81,82,84–86,88,91,92], unspecified-type troponin [63,67,80,82], CK-MB [14,18,34,37,40,45,46,49,52,57,59,62,66,68,70,74–76,78–82,84,87–90,92], BNP [14], and NT-proBNP [49,52,61,63,75,85,87,88]. Leukocytosis, usually neutrophil dominant, was a prominent finding in all investigations which reported white blood cell count [14,34,37,38,40–42,47,64–66,70,72–74,76,80,83,84,86,87,89]. One study reported hemoglobin and plasma protein to be higher in patients with pulmonary edema [39]. Elevated serum glucose [26,34,37,38,41,47,61,62,65,66,72,73,84,85,87], amylase [37,38,44,47,62,81,84,87], creatinine phosphokinase [18,26,38,44,47,51,55,59,62,69,72,74,76,78,90], lactate dehydrogenase [55,78,81,89,90], alanine transaminase [73,83,86,87], aspartate transaminase [26,44,62,64,73,83,86,87,90], and C-reactive protein [42,50,87] were other laboratory findings. Interleukin-8, a chemotactic cytokine, was found to be high and correlate with severity of cardiac dysfunction in one study [55].

### Diagnostic investigations

Several studies used pulmonary artery catheterization to evaluate the hemodynamic parameters [35,36,39,43,56,75,90]. Stroke volume index and cardiac index were decreased and pulmonary artery occlusion pressure was elevated among all cases in these studies resembling the profile of acute congestive heart failure. Nouira et al. [56] have found that just like LV, RV function gets impaired in scorpion myocarditis.

Echocardiography findings in the studies included regional or global hypokinesia [13,26,34,36–38,45,48,51,52,54,57,59,61,62,67,71,72,75–77,79,81,83–87,90,91], chamber dilation [45,53,59,68,69,73,76,80], decreased left ventricular ejection fraction (LVEF) [13,14,18,26,34,36–41,45,46,48,49,51–55,57–75,77–93], decreased fractional shortening (FS) [37,38,41,48,49,51,53,55,62,73,80,92], right ventricular (RV) dysfunction [56,60,90], and mitral or tricuspid regurgitation [13,26,34,36,37,45,48,51,57,73,75–77,79,80,82,83,87,89].

Four studies investigated histopathological findings in the autopsy of the patients [38,44,47,50]. Interstitial edema [38,44,50], dilation of chambers [38,44], inflammatory infiltrates such as monocytes [50], lymphocytes [50], polymorphonuclear cells, and eosinophils [44,47,50], increased left ventricular wall thickness [44], foci of hemorrhage [44] and necrosis [47,50] and engorgement of myocardial vessels [38] were the histopathological findings.

Five studies investigated cardiac magnetic resonance (CMR) imaging in scorpion envenomation [63,67,75,81,85]. Enhancement of myocardium in T1-sequence and mild intramyocardial late enhancement (indicative of irreversible damage) were reported by Lonati et al. [81]. Another study [75] reported global LV hypokinesia and impaired systolic function with diffuse myocardial edema. Additionally, Miranda et al. [85] reported apical ballooning and global edema of the midmyocardium in the acute phase of envenomation. Abroug et al. [63] and Ben Jemaa et al. [67] reported instances of basal ballooning.

Nuclear imaging to investigate myocardial perfusion was used in four studies [26,27,46,87]. A significant topographical correlation was found between wall motion abnormalities and the perfusion change in three [26,27,46], but one reported no ischemia [87]. In these studies, the perfusion of apical segments was less affected, and basal portions of the ventricular walls were more involved [26,27,46]. Hypoperfusion was also pronounced in interventricular septum [26,27]. One study used radionuclide ventriculography and multigated acquisition (MUGA) scan which estimate ventricular function [90].

### Treatment strategies

Several treatment strategies were presented in the literature. Scorpion antivenom (SAV) [36–38,42–44,49,55,56,61,62,65,66,69,74,75,78,80,81,84,85,87,88] was used in most of the studies. Tetanus prophylaxis in the form of tetanus toxoid was also used [42,65,87]. Standard heart failure treatment, including inotropes as well as diuretics to treat cardiogenic shock and pulmonary edema, was used in almost all studies. Dobutamine was the most widely used agent to treat shock in scorpion stings [13,14,18,26,35,36,38,40,41,43,45,46,48,49,56,58,61–64,67,68,71,73,78,80,81,83,84,86–89,92]. Mechanical ventilation was required in 35.2% (87 of 247) of cases to treat respiratory failure [14,18,26,34–38,40,43–45,47,57,61–71,73–83,85–92]. Digitalis was occasionally administered [37,40,44,47,54,69–71,83,90]. Antihistamines [47,68,74,87] and corticosteroids [36,47,56,64,66,68,69,74,75,77,78,81,83,87,89] were used in a number of research papers. Aspirin was used in three studies [64,66,74]. Nitrates such as nitroglycerine and sodium nitroprusside were used in several investigations, all of which reported pulmonary edema [18,56,58,64,66,74,76,87,92]. Prazosin [13,14,18,34,58,60,68,75,81,87] and doxazosin [42,49,65,88] were other used medications. In one case of refractory cardiogenic shock, an intra-aortic balloon pump was used as a means of rescue therapy with a desirable outcome [71].

### Outcome

The outcome of scorpion-related myocarditis was generally favorable, with a mortality rate of 7.3% (33 of 450). Almost all the studies that conducted serial echocardiography or hemodynamic studies to evaluate the progress of cardiac function reported either complete recovery (secondary LVEF>50%) [13,26,27,34–38,45,46,51,53,54,56–69,71,73–77,79–82,85–92] or significant improvement of LVEF in survivors [66]. Even CMR findings were shown to be reverted to normal [63,85]. The reported sequela were dilated cardiomyopathy in one case [42], and suboptimal LVEF at discharge (45%) in another [66].

## Discussion

Scorpion envenomation is associated with numerous complications and is an established cause of acute myocarditis [19]. Myocardial damage is caused by a complex interplay of hemodynamic, metabolic, and myocardial variables; as a result, it is difficult to pinpoint the specific mechanism of venom’s effect on the heart [29].

Scorpion-related cardiac damage has been histopathologically proven to be actual myocarditis; inflammatory infiltrates, necrosis, and tissue edema observed in pathological investigations is compatible with histological findings of myocarditis [38,44,47,50,94,95].

Findings regarding cardiac magnetic resonance (CMR) in scorpion myocarditis are scarce. Enhancement in T1-sequence, sub-epicardial to intra-myocardial late gadolinium enhancement [81], global LV hypokinesia, and impaired systolic function with myocardial edema [75] found in CMR imaging of scorpion-stung patients are consistent with the CMR criteria of myocarditis [33]. Other findings of interest were apical [85] or basal ballooning [63,67], leading some to label the scorpion-related myocarditis as “inverted Takotsubo syndrome” [63]. Takotsubo cardiomyopathy is similarly caused by catecholamine excess and apical ballooning is one of its characteristic findings [63]. Perfusion scintigraphy has proven the presence of ischemia in severe scorpion envenomation [26,27,46].

The incidence of myocarditis among scorpion envenomation cases is challenging to assess. Most of the case series reported the number of admitted cases, not all emergency department visits due to scorpion sting, hence not taking the cases that do not need referral or admission into account. Also, the incidence of myocarditis depends on the scorpion species composition of the region, utilized diagnostic criteria, and how long after the sting the patients are assessed. Cardiac biomarkers and echocardiography findings might fluctuate fast following the sting, resulting in different findings [49]. The majority of cases reported in the literature were children. This is in line with previous studies that reported children are more likely to develop cardiorespiratory symptoms, including cardiogenic shock and pulmonary edema, than adults [16]. It has been demonstrated that if untreated, the younger the patient, the higher the fatality rate is [31]. This is probably due to the higher venom-to-body mass ratio in children. Abroug et al. reported that mortality due to acute heart failure and pulmonary edema in scorpionism is about 0.27% [22].

General manifestations like fever, sweating, and restlessness are the result of sympathetic excitation, catecholamine storm, and possibly pain, and they are not specific to cases with cardiac involvement [19]. Parasympathetic excitation is responsible for hypersalivation, miosis, diarrhea, vomiting, bradycardia, increased respiratory secretions, and priapism. These parasympathetic responses are less marked than sympathetic responses [19]. Hyperglycemia is common in severely envenomed individuals and can affect the balance of other electrolytes, namely causing hypokalemia [84]. One study found that cases without signs of myocarditis were more likely to have seizures, coma, abdominal pain, ileus, oliguria, and low blood pressure, while myocarditis cases were more likely to have pulmonary edema and respiratory distress [34]. Bouaziz et al. [96] reported that tachypnea could be present in cases without pulmonary edema too.

Regarding other cardiovascular presentations, we believe that since tachycardia is present in both cases with and without cardiac involvement [97], it can be attributed to the sympathetic excitation and catecholamine effects, as well as shock resulting from myocarditis. An early phase of hypertension usually exists, resulting from the catecholamine release; however, if myocardial stunning occurs, this will change into hypotension and shock. Hypotension and bradycardia, especially if early after the sting, may be because of parasympathetic excitation rather than shock [19,98].

Pulmonary edema has been reported to be present in 7%–46% of all scorpion stings [98]. The occurrence of this complication also depends on the composition of the scorpion species. For example, one study found that 22.7% out of 888 scorpion sting cases by the H. tamulus developed pulmonary edema [99]. Our study estimates that it happens in 60.7% of scorpion-related myocarditis cases. Multiple factors have been regarded as the origin of pulmonary edema in scorpion envenomation [38,44,100]. As discussed, scorpion venom, directly and indirectly, affects the myocardium. Furthermore, not only have numerous studies reported a decrease in LVEF, but also hemodynamics studies in both human and animal models have shown increased pulmonary arterial pressure and decreased stroke volume following scorpion envenomation [36,43,101–103]. One study found that the severity of pulmonary edema is correlated with EF decrease [54]. These findings are in favor of cardiac origin of pulmonary edema; however, some suggest that it may be of both cardiac and non-cardiac origin [26,42,76,100].

Some studies focused on the diagnostic significance of cardiac biomarkers in scorpion stings [49,52,55,58,60,61,97,104,105]. While high troponin is a diagnostic criterion for myocarditis, it may not always indicate myocardial dysfunction [104]. Several studies investigated the sensitivity for troponin levels to predict cardiac dysfunction using echocardiography as the gold standard, with mixed results. While some suggest excellent sensitivity and specificity of troponin for detecting cardiac dysfunction in scorpion envenomation [55,58,60,97], two studies argue otherwise [57,61]. One study points out that serial measurements should be done for troponin to gain sensitivity [61]. In general, the existence of myocardial dysfunction and the severity of the envenomation are well correlated with troponin levels making troponin a good screening tool especially [34,55]. Other studies showed that CK-MB is likely to be elevated in most envenomations (even those without cardiac dysfunction), while NT-proBNP and troponin are more specific markers of cardiac dysfunction [55,57,58,106]. Khalaf et al. [52] found low sensitivity and specificity for CK-MB [57]. Natriuretic peptides, BNP and NT-proBNP, were investigated in some studies with varied results [49,52,58,61,105]. While Gökay et al. [49] and Prasad et al. [58] showed a potential for NT-proBNP to predict myocardial dysfunction, Sofer et al. [61] found that it has low sensitivity and specificity.

The most frequent ECG patterns after sinus tachycardia were ST-T segment alterations and Q waves, which can be indicators of cardiac damage, ischemia, or strain in myocarditis [107]. These ECG patterns were shown to coincide with elevated cardiac biomarkers [108]. However, as seen in the results, all these patterns, including sinus tachycardia, have varying incidences. Sofer et al. [61] and Das et al. [93] reported abnormal ECG findings in cases without cardiac dysfunction, this suggests either the venom itself or its subsequent substance release or autonomic excitation can affect cardiac electrical conduction even in the absence of clinically-evident myocarditis. As the result of conduction disturbances, life-threatening arrhythmias such as ventricular tachycardia and ventricular fibrillation can occur and cause sudden deaths [84,109]. Another contributor to arrhythmias can be the glucose and electrolyte imbalance, thus appropriate management is advised. ECG in isolation seems to be neither a sensitive nor specific tool to diagnose scorpion-related myocarditis; however, it is still a valuable tool to detect life-threatening arrhythmias and can help the diagnosis of myocarditis when combined with other diagnostic criteria.

Echocardiographic findings are among the diagnostic criteria of clinical myocarditis, and as expected, typical changes are present in the reported literature [33], most notably hypokinesia and diminished EF and FS in the majority of cases. One study [61] reported normal echocardiography in the presence of clinical manifestations of heart failure [93]. This lends credence to the theory that pulmonary edema, which is usually regarded as a classic sign of congestive heart failure, may sometimes be of extra-cardiac origin in scorpion envenomation [100]. Additionally, Das et al. [93] also found echocardiographic changes in four patients in the absence of clinical evidence of myocarditis, showing that scorpion myocarditis can sometimes be subclinical. Sofer et al. [61] found that out of the 50 patients with normal echocardiography performed within 3 hours of hospital arrival, none had developed any abnormality in repeat echocardiography after 24 hours, making echocardiography an excellent diagnostic and prognostic tool.

Numerous treatment strategies have been implemented in scorpion stings including SAV, tetanus toxoid, analgesia, prazosin, inotropic agents, digoxin, and prazosin, with various levels of evidence. We discuss the treatment strategies focused on myocarditis. Analgesia is recommended in all cases of the sting, however using opioids to treat scorpion envenomation with evidence of systemic toxicity is discouraged by some, particularly in pediatrics [110,111]. Regarding immunosuppressive therapy, corticosteroids were used in some studies; however, their efficacy remains unclear [33].

Routine administration of SAV in all cases of scorpion envenomation is the subject of debate, and its benefit in severe cases of envenomation is even more uncertain [19,112]. It is suggested that since SAV acts by binding to the toxin, its administration may not be effective after systematic responses like catecholamine release, pulmonary edema, and shock have set in [19]. In addition to the delay of SAV administration, it is also necessary to take into account its neutralization titer; some studies show that the antivenom is effective provided that the neutralization titer of the antivenom is high, in practice greater than 20 Effective Dose 50 (ED50) per mL [111,113]. In conclusion, we do not rule out the benefit of SAV since there exist many studies (mostly observational) that rule in favor of SAV. The study by Pandi et al., showed that utilization of SAV plus prazosin improves recovery time and decreases the incidence of myocardial dysfunction in scorpion envenomation when compared to the use of prazosin alone [114]. Similarly, Kumar et al. [18] found that early administration of SAV and prazosin reduces the risk of myocardial dysfunction. A point to remember is that the main objective of SAV is to eliminate the venom from the body, including from deep tissues, by a mechanism of mass action [115]. The SAV must be used in conjunction with symptomatic and supportive treatment to cure the envenomation and its sequelae.

The mainstay of scorpion-related myocarditis management is anti-heart failure treatment. The use of inotropes and diuretics suggested by guidelines in intensive care units with respiratory and mechanical cardiopulmonary support facilities is recommended [33]. An important exception to the cardiogenic shock guidelines may be the superiority of dobutamine compared to vasopressors such as norepinephrine and epinephrine, since norepinephrine and epinephrine already exist at high levels in catecholamine storm induced by scorpion venom. Dobutamine increases the contractility of the heart but has a minimal net effect on vascular resistance [116]. A feature that is most useful in the setting of an adrenergic cardiomyopathy like scorpion-related myocarditis. It is reported to result in the improvement of hemodynamic parameters [96,102,103]. Studies have shown that cardiac output was increased and peripheral vascular resistance was decreased after administration of dobutamine in scorpion envenomation [43,96,102,103]. Nitroglycerin, a vasodilator that decreases preload and afterload, has been suggested for treating pulmonary edema [19]. Prazosin is also used for the treatment of cardiovascular complications of scorpion envenomation [117–119]. Prazosin decreases preload and afterload, decreases the sympathetic outflow of the central nervous system, and promotes insulin secretion, hence antagonizing the scorpion venom’s hemodynamic and metabolic effects [118]. It has also shown promise in treating pulmonary edema caused by scorpion sting [120,121]. Prazosin is currently recommended in the treatment of scorpion envenomation, especially if symptoms of excess catecholamines, such as hypertension, are evident [19,118]. However, this therapy is contraindicated in shock state [71]. Exploring additional options that maintain perfusion and support cardiac function until the acute phase of envenomation wears off can prove beneficial. As seen in one study, using intra-aortic balloon pump in one case with an initial EF of 10% and refractory shock, hemodynamic optimization, and later complete recovery of cardiac function were achieved [71].

Administering respiratory support or oxygen therapy in the form of non-invasive or mechanical ventilation as the first cardiac and respiratory symptoms appear, is of utmost importance. Many patients die of hypoxemia caused by respiratory failure in scorpion envenomation. Mechanical ventilation should not only be considered in respiratory distress caused by pulmonary edema, but also in cases of refractory shock, severe transient encephalopathy, hypopnea, and apneic episodes that sometimes occur in severe envenomation [61].

Our finding of 7.3% mortality is important, considering this disease usually afflicts children and young patients with previously good health status. In survivors LVEF usually reverts back to normal, indicating that scorpion-related myocarditis is probably associated with less complications than clinically-evident viral myocarditis [122]. Bawaskar and Bawaskar [123,124] reported that the severity of cardiovascular symptoms varies with the victim’s age, body mass, season, the amount of poison injected, and the length of time the person was envenomed. Prasad and Mishra [14] reported that tachypnea, myocarditis, shock, encephalopathy, and the development of acute pulmonary edema were shown to be independently associated with a poor prognosis in patients with scorpion envenomation, while with little or minimal organ involvement, the outcome of scorpion stings are excellent. The time from the sting to arrival at the hospital is also a factor [14]. Similarly, Bahloul et al. found that coma, convulsions, pulmonary edema, and cardiogenic shock are factors associated with poor prognosis [16,28].

Because of the distinct pathophysiology of scorpion venom-associated myocarditis, future research should focus on providing a uniform guideline to standardize treatment. The efficacy of SAV and adequate dosage in patients who develop myocarditis should be further investigated.

### Clinical take-home message

When encountering patients with severe scorpion envenomation, physicians should keep the culprit species when available or the species composition of the area in mind to predict which complications to expect. While general symptoms may appear as early as minutes after the sting, cardiopulmonary symptoms, take a few hours to present [61,62,72,125]. Pulmonary edema and shock develop later than tachycardia or hypertension so continuous surveillance is recommended [125]. Obtaining ECG, serial serum cardiac biomarkers and chest x-ray are recommended in patients with signs and symptoms of systemic envenomation to evaluate the presence of cardiac damage and pulmonary edema. Elevated cardiac biomarkers, ECG findings like ST-T changes, and pulmonary edema are associated but not always indicative of cardiac dysfunction so they should be correlated with close clinical evaluation and echocardiography findings to guide therapy. Echocardiography in the presence of elevated cardiac biomarkers, ECG changes, pulmonary edema, or shock is recommended when available. In the conditions of such presentations, in addition to ICU care, routine laboratory investigations, pain management, and glucose, electrolyte, and blood gas evaluation, we recommend that patients be oxygen and heart monitored and cardiopulmonary resuscitation equipment be kept at hand.

Supportive therapy to address any cardiopulmonary complication should be initiated. Mechanical or non-invasive oxygen therapy in respiratory failure or impending respiratory collapse is imperative. Hypertension and symptoms of catecholamine excess can be managed with prazosin. Shock and hypotension are most probably of cardiac origin hence they should be managed according to the guidelines of cardiogenic shock therapy. However the use of norepinephrine and epinephrine is discouraged. Dobutamine is the most widely-used inotrope in these patients. Nitroglycerine, diuretics, and prazosin can be used to treat pulmonary edema if hemodynamic status is stable. In severe forms of envenomation (with systemic manifestations and/or cardiac dysfunction), the SAV in conjunction with symptomatic and supportive treatment to cure the envenomation and its sequelae is strongly advised.

Venom-induced and antivenom-induced anaphylaxis should be considered in these patients since some symptoms of anaphylaxis can overlap with symptoms of severe systemic envenomation [126–129]. Furthermore, adrenergic storm in severe envenomations can mask the symptoms of anaphylaxis or even protect against it [126,130]. Cutaneous signs (generalized pruritus, urticaria, and rash), eosinophilic leukocytosis, and quick onset of symptoms (within minutes) are in favor of anaphylaxis [129]. To diagnose antivenom-associated anaphylaxis, it is essential to look for signs of anaphylaxis precisely before administering the antivenom.

With proper supportive management, in most uncomplicated cases, clinical improvement starts soon and regression of pulmonary edema and shock is expected to occur within 48 to 96 hours. Significant reversal of LV dysfunction on echocardiogram or hemodynamic study is achieved roughly within one week [34,36,62].

### Limitations

Our study is not free of limitations. First, as with any other systematic review, there may be studies and citations that were not included in our study despite the use of the PRISMA protocol and a comprehensive database search. Second, we narrowed our inclusion criteria to myocarditis cases while there may be other cardiac complications related to a scorpion sting. Some studies only counted scorpion-sting patients admitted to the intensive care unit, which may have led to an overstatement of some presentations and fatalities. Another important limitation is that our study is based on case reports or series, making it impossible to draw firm conclusions. Also, due to publication bias, mild or moderate cases may have remained neglected in the literature.

## Conclusion

Myocarditis associated with scorpion envenomation is a rare but potentially devastating complication of scorpion stings which is more common in children. Although scorpion envenomation usually causes mild to moderate symptoms, myocarditis should remain on the list of differential diagnoses in the case of cardiopulmonary presentations. Screening with cardiac markers and echocardiography can help the diagnosis. The management of cardiogenic shock and pulmonary edema should be the main goal of treatment. With proper and timely management, the outcome could be favorable.

## Supporting information

S1 Table PRISMA Checklist. (DOCX)

S2 Table Search strategies. (DOCX)

S3 Table Excluded articles after full-text review and the reason for exclusion. (DOCX)

S4 Table Description of included case reports. (DOCX)
