# Supplementary material for: Scorpion envenomation-associated myocarditis: A systematic review
Source: PLoS Negl Trop Dis. 2023 Apr 5;17(4):e0011219. doi: 10.1371/journal.pntd.0011219 (PMC10075437; doi:10.1371/journal.pntd.0011219)
Supplement: S2 Table — (DOCX) [file pntd.0011219.s002.docx]

| **S2 Table. Search strategies** | |
| --- | --- |
| Databases | Search Strategies |
| PubMed | ((Scorpions[MeSH Terms]) OR (Scorpion Stings[MeSH Terms]) OR (Scorpion Venoms[MeSH Terms]) OR (scorpion*[Title/Abstract])) AND ((Myocarditis[MeSH Terms]) OR (carditis[Title/Abstract]) OR (Cardiomyopathies[MeSH Terms]) OR ("Cardiomyopath*"[Title/Abstract]) OR ("myocard*"[Title/Abstract])) |
| Scopus | (INDEXTERMS("scorpion*") OR TITLE-ABS-KEY("scorpion*")) AND (INDEXTERMS(myocarditis) OR TITLE-ABS-KEY("carditis" OR "myocard*" OR "cardiomyopath*")) |
| Web of Science | (TS=("scorpion*")) AND (TS=("carditis" OR "myocard*" OR "cardiomyopath*")) |
| Google Scholar | “scorpion*” AND “myocard*” |
