# Supplementary material for: Scorpion envenomation-associated myocarditis: A systematic review
Source: PLoS Negl Trop Dis. 2023 Apr 5;17(4):e0011219. doi: 10.1371/journal.pntd.0011219 (PMC10075437; doi:10.1371/journal.pntd.0011219)
Supplement: S3 Table — (DOCX) [file pntd.0011219.s003.docx]

| **S3 Table. Excluded articles after full-text review and the reason for exclusion** | |
| --- | --- |
| Author and year | Reason for exclusion |
| Aboumaâd et al., 2014 (1) | No clear definition of myocarditis and grouping the patients with myocardial damage with other organ failure cases. |
| Aghabiklooei et al., 2013 (2) | Myocardial infarction is suggested as the cause. |
| Ahmed et al., 2018 (3) | No clear definition of myocarditis and data regarding the myocarditis cases are not reported separately. |
| Alpay et al., 2008 (4) | Clinical symptoms of interest are absent. |
| Angral et al., 2010 (5) | Have neither echocardiography nor cardiac marker. |
| Arivoli and Ganesh, 2015 (6) | Grouping the patients with myocardial damage with other organ failure cases. |
| Barzilay et al., 1982 (7) | Myocardial infarction is suggested as the cause. |
| Bawaskar, 1982 (8) | Have neither echocardiography nor cardiac marker. |
| Bawaskar and Bawaskar, 1989 (9) | Have neither echocardiography nor cardiac marker. |
| Bawaskar and Bawaskar, 1991 (10) | Have neither echocardiography nor cardiac marker. |
| Bawaskar and Bawaskar, 1991 (11) | Have neither echocardiography nor cardiac marker. |
| Bawaskar and Bawaskar, 1992 (12) | Have neither echocardiography nor cardiac marker. |
| Bawaskar and Bawaskar, 1994 (13) | Have neither echocardiography nor cardiac marker. |
| Bawaskar et al., 2014 (14) | Acute myocardial infarction is suggested as the cause. |
| Bawaskar et al., 2022 (15) | Presentation could be attributed to arrhythmia rather than myocarditis. Normal echocardiogram. |
| Benvenuti et al., 2002 (16) | Histological investigation in favor of ischemic myocardial damage. No echocardiography or cardiac marker. |
| Bhadani, 2006 (17) | Have neither echocardiography nor cardiac marker. |
| Bhoite et al., 2015 | Have neither echocardiography nor troponin. |
| Biswal et al., 2006 (18) | Data pertaining to myocardial damage cases not separable from other cases. |
| Blum et al., 2000 (19) | Normal echocardiography and no cardiopulmonary symptoms. |
| Bosnak et al., 2009 (20) | Grouping the patients with myocardial damage with other organ failure cases. |
| Bouaziz et al., 2006 (21) | Data pertaining to myocardial damage cases not separable from other cases. |
| Bouaziz et al., 2008 (22) | Have neither echocardiography nor cardiac marker. |
| Bouaziz et al., 2020 (23) | Data could not be unambiguously attributed to those with myocarditis. |
| Chakroun-Walha et al., 2018 (24) | No clear definition of myocarditis and data regarding the myocarditis cases are not reported separately. |
| Das et al., 1995 (25) | Definition of myocarditis differs from our criteria, and data pertaining to the cases which do fulfill our criteria are not separable from other cases |
| Devarbhavi et al., 2013 (26) | Data pertaining to myocardial damage cases not separable from other cases. |
| Dudin et al., 1991 (27) | Have neither echocardiography nor cardiac marker. Data pertaining to myocardial damage cases not separable from other cases. |
| El-Asheer et al., 2019 (28) | Data pertaining to myocardial damage cases not separable from other cases. |
| El-Deek et al., 2017 (29) | Does not present data of our interest. |
| Elatrous et al., 1999 (30) | Data could not be unambiguously attributed to those who fulfill the myocarditis criteria. |
| Garg et al., 1983 (31) | Have neither echocardiography nor cardiac marker. |
| Gokdemir and Sezer, 2013 (32) | Echocardiography did not demonstrate cardiac dysfunction in any of the subjects. Troponin was negative in all cases. |
| Gueron et al., 1967 (33) | Have neither echocardiography nor cardiac marker. |
| Gueron and Yaron, 1970 (34) | Have neither echocardiography nor cardiac marker. |
| Gupta et al., 2009 (35) | Data pertaining to myocardial damage cases not separable from other cases. |
| Gürses et al., 2018 (36) | Data pertaining to the scorpion sting cases not separable from other myocarditis cases. |
| Horoz et al., 2019 (37) | Data pertaining to myocardial damage cases not separable from other cases. |
| Isbister and Bawaskar, 2014 (38) | Review article with no presented case. |
| Karnad et al., 1997 (39) | Have neither echocardiography nor cardiac marker |
| Kayikcioglua et al., 2006 (40) | Diagnosis of myocarditis is uncertain. Acute myocardial infarction is suggested. |
| Kumar et al., 2012 (41) | The scorpion sting was not confirmed. |
| Kumaryadav et al., 2020 (42) | No clear definition or diagnostic criteria for myocarditis. |
| Madan and Rao, 1978 (43) | Have neither echocardiography nor cardiac marker |
| Mahadevan et al.,1981 (44) | Have neither echocardiography nor cardiac marker |
| Meki et al., 2002 (45) | Duplicate cases. Grouping the myocarditis cases with other cases and different criteria for myocarditis. |
| Mishra and Prasad, 2015 (46) | Editorial piece with no case presentation. |
| Murthy et al.,1991 (47) | Have neither echocardiography nor cardiac marker. |
| Narayanan et al., 2006 (48) | Have neither echocardiography nor cardiac marker. |
| O’Connor et al., 2017 (49) | Cause of decreased cardiac function, could be interpreted as acute decompensated heart failure. |
| Pandi et al., 2014 (50) | Data pertaining to myocardial damage cases not separable from other cases. |
| Patra et al., 2013 (51) | Cause of decreased cardiac function was myocardial infarction, proved by coronary angiography. |
| Pol et al., 2011 (52) | No clear definition or diagnostic criteria for myocarditis. |
| Razi and Malekanrad, 2008 (53) | Have neither echocardiography nor cardiac marker. |
| Reddy et al. (54) | Troponin normal and echocardiography did not show any features suggestive of myocarditis. |
| Sagarad et al., 2013 (55) | Duplicate cases. |
| Sagarad et al., 2013 (56) | Duplicate cases. |
| Santhanakrishnan et al., 1986 (57) | Have neither echocardiography nor cardiac marker. |
| Sengupta et al., 2009 (58) | Have neither echocardiography nor cardiac marker. |
| Shashidhar et al, 2014 (59) | Have neither echocardiography nor cardiac marker. |
| Singhal et al, 2009 (60) | Have neither echocardiography nor cardiac marker. |
| Sofer et al. 1991 (61) | Cardiac marker is not troponin. |
| T. Poon-King et al, 1963 (62) | Have neither echocardiography nor cardiac marker. |
| Tomlinson and Elston, 2005 (63) | Review article with no presented case. |
| Umapathi et al., 2020 (64) | Diagnostic criteria of myocarditis are not defined. Have neither echocardiography nor cardiac marker. |
| Vaucel et al., 2020 (65) | Have neither echocardiography nor cardiac marker |
| Verma et al., 2021(66) | Diagnosis of myocarditis is uncertain. Acute myocardial infarction is suggested. |
| Yarom et al., 1970 (67) | Review article with no presented case. |
| Zouizra et al., 2020 (68) | Cardiac involvement is endocarditis. |

**References**

1. Aboumaad B, Lahssaini M, Tiger A, Benhassain SM. Clinical comparison of scorpion envenomation by Androctonus mauritanicus and Buthus occitanus in children. Toxicon. 2014;90:337-43.

2. Aghabiklooei A, Zamani N, Hassanian-Moghaddam H. Getting stung by black scorpion Androctonus crassicauda: a case report. Hum Exp Toxicol. 2014;33(10):1081-4.

3. Ahmed AE, Hassan MH, Rashwan NI, Sayed MM, Meki AMA. Myocardial injury induced by scorpion sting envenoming and evidence of oxidative stress in Egyptian children. Toxicon. 2018;153:72-7.

4. Alpay NR, Satar S, Sebe A, Demir M, Topal M. Unusual presentations of scorpion envenomation. Human and Experimental Toxicology. 2008;27(1):81-5.

5. Angral R, Lachala S, Gupta S, Ahmed A, Kundan S. Hemodynamic and myocardial manifestations of poisoning by Indian scorpion (Mesobuthus tamulus). J Anaesthesiol Clin Pharmacol. 2010;26(1):117-8.

6. Arivoli K, Ganesh J. A STUDY ON THE CLINICAL PROFILE OF SCORPION ENVENOMATION IN CHILDREN. J Evol Med Dent Sci-JEMDS. 2015;4(90):15522-6.

7. Barzilay Z, Shaher E, Schneeweiss A, Motro M, Shem-Tov A, Neufeld HN. Myocardial damage with life-threatening arrhythmia due to a scorpion sting. Eur Heart J. 1982;3(2):191-3.

8. Bawaskar HS. Diagnostic cardiac premonitory signs and symptoms of red scorpion sting. Lancet. 1982;1(8271):552-4.

9. Bawaskar HS, Bawaskar PH. Stings by red scorpions (Buthotus famulus) in maharashtra state, india: A clinical study. Transactions of the Royal Society of Tropical Medicine and Hygiene. 1989;83(6):858-60.

10. Bawaskar HS, Bawaskar PH. Cardiovascular manifestations of severe scorpion sting in India (review of 34 children). Ann Trop Paediatr. 1991;11(4):381-7.

11. Bawaskar HS, Bawaskar PH. Scorpion sting: a review of 121 cases. Journal of Wilderness Medicine. 1991;2(3):164-74.

12. Bawaskar HS, Bawaskar PH. Management of the cardiovascular manifestations of poisoning by the Indian red scorpion (Mesobuthus tamulus). Br Heart J. 1992;68(5):478-80.

13. Bawaskar HS, Bawaskar PH. Vasodilators: scorpion envenoming and the heart (an Indian experience). Toxicon. 1994;32(9):1031-40.

14. Bawaskar HS, Bawaakar PH, Bawaskar PH. Severe scorpion envenoming results in acute myocardial infarction. Journal of Cardiovascular Disease Research. 2014;5(3):27-9.

15. Bawaskar HS, Bawaskar PH, Bawaskar PH. Stung to the Heart. JACC: Case Reports. 2022;4(1):54-8.

16. Benvenuti LA, Douetts KV, Cardoso JL. Myocardial necrosis after envenomation by the scorpion Tityus serrulatus. Trans R Soc Trop Med Hyg. 2002;96(3):275-6.

17. Bhadani UK, Tripathi M, Sharma S, Pandey R. Scorpion sting envenomation presenting with pulmonary edema in adults: a report of seven cases from Nepal. Indian J Med Sci. 2006;60(1):19-23.

18. Biswal N, Bashir RA, Murmu UC, Mathai B, Balachander J, Srinivasan S. Outcome of scorpion sting envenomation after a protocol guided therapy. Indian J Pediatr. 2006;73(7):577-82.

19. Blum A, Jawabreh S, Gumanovsky M, Soboh S. Scorpion envenomation and myocardial damage. Isr Med Assoc J. 2000;2(4):318-9.

20. Bosnak M, Ece A, Yolbas I, Bosnak V, Kaplan M, Gurkan F. Scorpion sting envenomation in children in southeast Turkey. Wilderness Environ Med. 2009;20(2):118-24.

21. Bouaziz M, Bahloul M, Hergafi L, Kallel H, Chaari L, Ben Hamida C, et al. Factors Associated with Pulmonary Edema in Severe Scorpion Sting Patients – A Multivariate Analysis of 428 Cases. Clin Toxicol. 2006;44(3):293-300.

22. Bouaziz M, Bahloul M, Kallel H, Samet M, Ksibi H, Dammak H, et al. Epidemiological, clinical characteristics and outcome of severe scorpion envenomation in South Tunisia: multivariate analysis of 951 cases. Toxicon. 2008;52(8):918-26.

23. Bouaziz M, Ben Hamida C, Chelly H, Bahloul M, Kallel H. Dobutamine in the treatment of severe scorpion envenoming. Toxicon. 2020;182:54-8.

24. Chakroun-Walha O, Karray R, Jerbi M, Ben Rebeh A, Jammeli C, Bahloul M, et al. Value of troponin levels in the diagnosis of cardiac dysfunction in moderate scorpion envenomation. Hum Exp Toxicol. 2018;37(6):580-6.

25. Das S, Nalini P, Ananthakrishnan S, Ananthanarayanan PH, Balachander J, Sethuraman KR, et al. Scorpion Envenomation In Children In Southern India. J Trop Med Hyg. 1995;98(5):306-8.

26. Devarbhavi PK, Murthy V, Al-Dubai SA, Alshagga MA. Clinical features and complications of scorpion sting: A descriptive study. Research Updates in Medical Sciences. 2013;1(2):16-20.

27. Dudin AA, Rambaud-Cousson A, Thalji A, Juabeh, II, Abu-Libdeh B. Scorpion sting in children in the Jerusalem area: a review of 54 cases. Ann Trop Paediatr. 1991;11(3):217-23.

28. El-Asheer OM, Hammad EEM, Mohamad IL, Saad K, Aziz NA. A randomized comparative study between intravenous and intramuscular scorpion antivenom regimens in children. Toxicon. 2019;159:45-9.

29. El-DeeK SEM, Sayed AA, Nassar AY, Mohey-Eldeen ZM, Eldeeb HM, Meki ARMA. Role of some vasoactive mediators in scorpion envenomed children: Possible relation to envenoming outcome. Toxicon. 2017;127:77-84.

30. Elatrous S, Nouira S, Besbes-Ouanes L, Boussarsar M, Boukef R, Marghli S, et al. Dobutamine in severe scorpion envenomation: effects on standard hemodynamics, right ventricular performance, and tissue oxygenation. Chest. 1999;116(3):748-53.

31. Garg AK, Pimparkar AB, Abraham P, Chikhalikar AA. Myocarditis and pulmonary edema following scorpion bite. (A case report). J Postgrad Med. 1983;29(1):46-8.

32. Gokdemir M, Sezer T. Evaluation of myocardial function using tissue Doppler imaging in children with moderate scorpion envenomation. Clin Toxicol (Phila). 2013;51(3):156-61.

33. Gueron M, Stern J, Cohen W. Severe myocardial damage and heart failure in scorpion sting. Report of five cases. Am J Cardiol. 1967;19(5):719-26.

34. Gueron M, Yaron R. Cardiovascular manifestations of severe scorpion sting. Clinicopathologic correlations. Chest. 1970;57(2):156-62.

35. Gupta BD, Parakh M, Purohit A. Management of Scorpion Sting: Prazosin or Dobutamine. J Trop Pediatr. 2009;56(2):115-8.

36. Gürses D, Oǧuz M, Yilmaz M. Clinical and echocardiographic evaluation of our patients with myopericarditis. Turk Klinikleri Pediatr. 2018;27(3):93-101.

37. Horoz OO, Yildizdas D, Aslan N, Gokay SS, Ekinci F, Erdem S, et al. Is there any relationship between initial hematological parameters and severity of scorpion envenomation? Turk J Pediatr. 2020;62(3):394-404.

38. Isbister GK, Sellors KV, Beckmann U, Chiew AL, Downes MA, Berling I. Catecholamine-induced cardiomyopathy resulting from life-threatening funnel-web spider envenoming. Medical Journal of Australia. 2015;203(7):302-4.

39. Karnad DR. Haemodynamic patterns in patients with scorpion envenomation. Heart. 1998;79(5):485-9.

40. Kayikcioglu M, Eroglu Z, Kosova B, Olukman M, Karatas A, Can LH, et al. Acute myocardial infarction following an arthropod bite: Is hereditary thrombophilia a contributing factor? Blood Coagulation and Fibrinolysis. 2006;17(7):581-3.

41. Kumar L, Naik SK, Agarwal SS, Bastia BK. Autopsy diagnosis of a death due to scorpion stinging - A case report. J Forensic Leg Med. 2012;19(8):494-6.

42. Yadav R, Alim M, Yadav Y, Singh D, Kumar A. Retrospective Study of Children with Scorpion Envenomation in a Tertiary Care Center of North India. Asia Pacific Journal of Medical Toxicology. 2020;9(3):91-6.

43. Madan MS, Rao L. Myocarditis from scorpion bite among children with review of the literature. Indian J Pediatr. 1978;45(371):381-5.

44. Mahadevan S, Choudhury P, Puri RK, Srinivasan S. Scorpion evenomation and the role of lytic cocktail in its management. The Indian Journal of Pediatrics. 1981;48(6):757-61.

45. Meki A, El-Deen ZMM, El-Deen HMM. Myocardial injury in scorpion envenomed children: Significance of assessment of serum troponin I and interteukin-8. Neuroendocrinol Lett. 2002;23(2):133-40.

46. Mishra O, Prasad R. Myocardial dysfunction in children with scorpion sting envenomation. Indian Pediatr. 2015;52(4):291-2.

47. Murthy KR, Shenoi R, Vaidyanathan P, Kelkar K, Sharma N, Birewar N, et al. Insulin reverses haemodynamic changes and pulmonary oedema in children stung by the Indian red scorpion Mesobuthus tamulus concanesis, Pocock. Ann Trop Med Parasitol. 1991;85(6):651-7.

48. Narayanan P, Mahadevan S, Serane VT. Nitroglycerine in scorpion sting with decompensated shock. Indian Pediatr. 2006;43(7):613-7.

49. O’Connor AD, Padilla-Jones A, Ruha AM. Severe bark scorpion envenomation in adults*. Clin Toxicol. 2018;56(3):170-4.

50. Pandi K, Krishnamurthy S, Srinivasaraghavan R, Mahadevan S. Efficacy of scorpion antivenom plus prazosin versus prazosin alone for Mesobuthus tamulus scorpion sting envenomation in children: a randomised controlled trial. Arch Dis Child. 2014;99(6):575-80.

51. Patra S, Satish K, Singla V, Ravindranath KS. Acute myocardial infarction following scorpion sting in a case with obstructive coronary artery disease. BMJ Case Rep. 2013;2013.

52. Pol R, Vanaki R, Pol M. The Clinical Profile and the Efficacy of Prazosin in Scorpion Sting Envenomation in Children of North Karnataka (India). J Clin Diagn Res. 2011;5(3):456-8.

53. Razi E, Malekanrad E. Asymmetric pulmonary edema after scorpion sting: a case report. Revista do Instituto de Medicina Tropical de São Paulo. 2008;50:347-50.

54. Reddy CR, Bompelli N, Khardenavis V, Deshpande A. Scorpion bite-induced ischaemic stroke. BMJ Case Rep. 2017;2017.

55. Sagarad SV, Kerure SB, Thakur B, Reddy SS, Balasubramanya K, Joshi RM. Echocardiography Guided Therapy for Myocarditis after Scorpion Sting Envenomation. J Clin Diagn Res. 2013;7(12):2836-8.

56. Sagarad SV, Thakur B, Reddy SS, Balasubramanya K, Joshi RM, Biradar-Kerure S. NT-proBNP in Myocarditis after a Scorpion Sting Envenomation. J Clin Diagn Res. 2013;7(1):118-21.

57. Santhanakrishnan BR, Gajalakshmi BS. Pathogenesis of cardiovascular complications in children following scorpion envenoming. Ann Trop Paediatr. 1986;6(2):117-21.

58. Sengupta S, Dhanapal P, Ravindran RD, Devi N. Cerebral blindness after scorpion sting. J Neuro-Ophthalmol. 2009;29(2):154-5.

59. Shashidhar G, Lokesh S, Karinagannanavar A. A Clinical Spectrum Of Scorpion Sting At Vijayanagar Institute Of Medical Sciences, Bellary. J Evol Med Dent Sci-JEMDS. 2014;3(57):12961-70.

60. Singhal A, Mannan R, Rampal U. Epidemiology, Clinical Presentation and Final Outcome of Patients with Scorpion Bite. J Clin Diagn Res. 2009;3(3):1523-8.

61. Sofer S, Shahak E, Slonim A, Gueron M. Myocardial injury without heart failure following envenomation by the scorpion Leiurus quinquestriatus in children. Toxicon. 1991;29(3):382-5.

62. Poon-King T. Myocarditis from scorpion stings. Br Med J. 1963;1(5327):374-7.

63. Elston DM. What's eating you? The South African fattail scorpion (Parabuthus transvaalicus). Cutis. 2005;76(5):299-300.

64. Umapathi K, Boopalan P, Maheshkumar VP, Ramamoorthy R. A study on clinical profile, management and outcome in pediatric patients admitted with scorpion envenomation. International Journal of Basic &amp; Clinical Pharmacology; Vol 9, No 10 (2020): October 2020DO - 1018203/2319-2003ijbcp20204096. 2020.

65. Vaucel J, Mutricy R, Hoarau M, Pujo JM, Elenga N, Labadie M, et al. Pediatric scorpionism in northern Amazonia: a 16-year study on epidemiological, environmental and clinical aspects. J Venom Anim Toxins Incl Trop Dis. 2020;26:e202000038.

66. Verma S, Mathew R, Khan KM, Gouthami V. Allergic myocardial infarction (Kounis syndrome) in a child with scorpion sting. Ann Pediatr Cardiol. 2021;14(3):441-2.

67. Yarom R, Gueron M, Braun K. Scorpion venom cardiomyopathy. Pathol Microbiol (Basel). 1970;35(1):114-7.

68. Zouizra Z, Benbakh S, Karimi SE, Boumzebra D. Tricuspid Valve Endocarditis Following a Scorpion Sting: A Case Report. World J Pediatr Congenit Heart Surg. 2020;11(3):374-6.
