# Supplementary material for: Scorpion envenomation-associated myocarditis: A systematic review
Source: PLoS Negl Trop Dis. 2023 Apr 5;17(4):e0011219. doi: 10.1371/journal.pntd.0011219 (PMC10075437; doi:10.1371/journal.pntd.0011219)
Supplement: S4 Table — (DOCX) [file pntd.0011219.s004.docx]

| **S4 Table: Description of included case reports** | | | | | | | |
| --- | --- | --- | --- | --- | --- | --- | --- |
| **Authors** | **Country** | **Year** | **Description of the case** | **ECG findings** | **Echocardiography findings** | **Treatment** | **Outcome** |
| **Abroug et al. (1)** | Tunisia | 2018 | A 36-year-old woman presented with pulmonary edema and shock.  Cardiac magnetic resonance (CMR) imaging showed basal ballooning of the left and right ventricles suggestive of an inverted biventricular Takotsubo syndrome. | Tachycardia with ST depression in four precordial leads | LVEF=30% | Continuous positive airway pressure, dobutamine | Survival with second normal CMR one month after recovery. |
| **Abroug et al. (1)** | Tunisia | 2018 | A 45-year-old woman aged 45 was admitted with pulmonary edema, shock, and positive troponin and NT pro-BNP. CMR showed basal ballooning. | Tachycardia, ST depression in the antero-septal precordial leads | LVEF=35% | Mechanical ventilation, dobutamine | Survival with normal LV function on repeat echocardiography. |
| **Agrawal et al. (2)** | India | 2015 | A 14-year-old male presenting with chest pain, cold sweating, and hypotension. Further work-up revealed pulmonary edema and positive troponin T. | ST-T changes and tachycardia | - | Intravenous dopamine, noradrenaline, dobutamine, furosemide and corticosteroids, oral aspirin, and nitrates, and noninvasive oxygen therapy with continuous positive airway pressure | Survival with normal EF but persistence of Q wave and T wave inversion |
| **Aslan et al. (3)** | Turkey | 2018 | A 7-year-old girl, stung by Leiurus quinquestriatus (aka Leiurus abdullahbayrami) presented with lethargy, normal blood pressure, and cold and swelled lower extremities. X-ray revealed bilateral pulmonary edema and extremely high troponin I levels. | Sinus tachycardia | LV systolic dysfunction with an initial EF of 56% reduced to 36% in following days | Tetanus vaccine, scorpion antivenom (SAV), doxazosin, dopamine, adrenaline | Survival with normal EF. |
| **Bayar et al. (4)** | Turkey | 2013 | A 49-year-old man with history of type 2 diabetes mellitus, and smoking.  A 49-year-old male presented with severe hypertension, tachycardia and manifestations of acute pulmonary edema. Positive troponin and CK-MB. | Peaked T waves, tachycardia | Diffuse impairment of the left ventricular systolic contractility with LVEF of less than 25% | Aspirin, low-molecular weight heparin, nitroglycerine, furosemide, and spironolactone anti-scorpion venom serum, and to counteract allergic reactions intravenous steroids, and antihistaminic therapy | Survival with marked improvement in LVEF (45%). |
| **Ben Jemaa et al. (5)** | Tunisia | 2021 | A 26-year-old man presented with acute pulmonary enema, shock state, and positive troponin. CMR showed LV systolic dysfunction predominantly in the basal segments with no late gadolinium enhancement, suggesting Takotsubo syndrome. | ST elevation in superior and ST depression in anterior and inferior leads | Impaired LV systolic function (LVEF=33%) with basal segment akinesia | Dobutamine | Echocardiographic control after 15 days showed recovery of normal LV systolic function. |
| **Bompelli et al. (6)** | India | 2018 | A 24-year-old female. 6 hours after an Indian red scorpion sting. Severe shortness of breath, perspiration. Tachycardia, tachypnea, hypotension and low oxygen saturation. Raised CK-MB. CXR showed unilateral right-sided haziness of the entire lung field. | Severe tachycardia, ST-T changes | Dilated hypocontractile LV with significant systolic, diastolic dysfunction | Dobutamine and noradrenaline intravenous infusion, analgesics, antihistaminic, topical xylocaine, prazosin, hydrocortisone, furosemide | Survival after complete resolution of symptoms and normal follow-up CXR and echocardiography. |
| **Brand et al. (7)** | Israel | 1988 | A 7-year-old girl, admitted with confusion, restlessness, sweating, vomiting, her heart rate was regular but high, pulmonary edema and shock state. | Diffuse ST-T change and ST depression in inferolateral leads, prolonged QTc interval, Repeat ECGs showed increase in QRS voltage in precordial leads, new Q waves, ST elevation and tall T in inferolateral leads | Dilated poorly contracted ventricles resembling dilated cardiomyopathy, gradual thickening of the LV wall in subsequent echocardiography | Hydrocortisone, SAV, digoxin, furosemide, prednisolone | Survival. Four months after discharge, echocardiography was back to normal. |
| **Bucaretchi et al. (8)** | Brazil | 2016 | A 44-year-old woman with a previous diagnosis of low-risk essential thrombocytopenia, stung by Tityus serrulatus, developed vomiting, pallor and confusion, followed by shock and loss of consciousness. Lab data showed leukocytosis, and elevated CK-MB and troponin. Brain CT scan revealed multiple, extensive brain infarcts including diffuse bilateral cerebellar hypodensity with partial involvement of both occipital lobes and thalamus, obstructive hydrocephaly with signs of cerebrospinal fluid extravasation and ascending transtentorial herniation. | ST depression | LVEF=47% | SAV, fluids replacement, dopamine, tracheal intubation and mechanical ventilation, midazolam, fentanyl, noradrenaline, enoxaparin | Expired due to extensive brain damage. |
| **D’sa et al. (9)** | India | 2015 | A 32-year-old woman presented with dyspnea, and palpitations after being stung by Hottentotta tamulus (old name: Mesobuthus tamulus). She had five episodes of cardiac arrest which were successfully resuscitated. Due to persistence of shock, intra-aortic balloon pump was inserted which resulted in improvement in her condition. | Three pulseless ventricular tachycardia/ventricular fibrillation episodes and one asystole which lead to cardiac arrest | EF=10%, global hypokinesia, raised left atrial filling pressure | Cardiopulmonary resuscitation, mechanical ventilation, dopamine, therapeutic hypothermia, epinephrine, norepinephrine, vasopressin, dobutamine, digitalis, intra-aortic balloon pump, milrinone | Although she showed features of hypoxic encephalopathy, she improved and returned to her daily activities in 30 days. Follow-up EF=58%. |
| **Dias et al. (10)** | India | 2012 | A 22-year-old man stung by a black scorpion, was well until 4 days later he sustained trauma close to the sting site. On the next day he developed vomiting and dizziness. He developed severe hypotension but pulse rate was 60 beats per minute. | T inversion in III and V1, biphasic T wave in V2 – V4, sinus bradyardia | Global hypokinesia,  LVEF=39% | Norepinephrine, atropine | Survival with normalized echocardiogram |
| **Dokur et al. (11)** | Turkey | 2017 | An 11-year-old-boy stung by Leiurus abdullahbayrami, developed confusion, tachycardia, hypotension, and dyspnea. CXR revealed pulmonary edema. In lab data leukocytosis was found. | - | Dilated LV, 1st degree mitral and tricuspid insufficiency secondary to annulus dilatation, FS=17.08%, EF=35.63% | SAV, dobutamine, oxygen with mask furosemide, enalapril, digoxin, morphine, acetylsalicylic enoxaparin, and pentoxifyllin | Survival with normal control echocardiography. |
| **Ersoy et al. (12)** | Turkey | 2016 | A 30-year-old male, with high blood pressure and heart rate, high CK-MB and troponin, dark brown urine. Coronary angiography was normal. | Sinus tachycardia | LVEF=45%, mild LV systolic dysfunction | IV fluid, scorpion antivenom,  acetylsalicylic acid, low molecular weight heparin, and nitroglycerin, IV steroid, antihistamines | Survival with normal EF (55%). |
| **Ismail et al. (13)** | Qatar | 2016 | A 24-year-old Nepalese male, sudden onset of nausea, vomiting, dizziness and profuse sweating, dyspneic. He was hypotensive, and tachycardic. Troponin T, CK-MB and pro BNP were elevated. CXR showed moderately severe lung congestion. CMR revealed global LV hypokinesia and systolic dysfunction with global myocardial edema suggestive of diffuse myocarditis; no evidence of myocardial scar by delayed gadolinium enhancement views. Coronary angiography was normal for both left and right coronary arteries. | sinus tachycardia with diffuse ST segment depression, short runs of ventricular tachycardia, prolongation of QT interval | Moderate to severe left ventricular global hypokinesia and impaired systolic function (EF=35%) with mild mitral regurgitation | SAV, IV fluids, mechanical ventilation, noradrenaline, dopamine, and maintenance doses of IV hydrocortisone and antibiotics (piperacillin plus tazobactam), prazosin | Survival with normal LV systolic function (EF=55%). |
| **Izquierdo and Buitrago (14)** | Colombia | 2012 | A 12-year-old boy, stung by Tityus pachyurus, presented with pain, several episodes of emesis which evolved to hematemesis, diaphoresis, mild respiratory distress, hypertension and tachycardia. He had cardiac arrest with pulseless ventricular tachycardia. He was successfully resuscitated, but sfter that developed hypotension, pulmonary edema and dysautonomia Elevated troponin I and CK-MB was appreciated in lab data. | - | Abnormal movement of interventricular septum, mitral insufficiency, dilatation of the LV | SAV, sodium nitroprusside, prazosin, cardiac resuscitation, dopamine | Survived with no sequelae. |
| **Jain et al. (15)** | India | 2006 | A 35-year-old male was stung by a brown colored Scorpion. He developed uneasiness, excessive sweating, 3-4 episodes of vomiting and inability to sustain posture in the standing position. On examination, he was drowsy and his extremities were cold. His pulse was 102 bpm and blood pressure was 90/80mmHg. Lung fields were clear. Neurological examination revealed mild right sided hemiparesis, extensor plantar response and left sided cerebellar signs. | Sinus tachycardia, Q waves and ST elevation in V_1_-V_3_ | Hypokinesia of interventricular septum, apex and apicolateral wall with moderate mitral regurgitation and reduced EF (31%) | Steroids and inotropic drugs | ECG changes and echocardiography became completely normal. Neurological signs and symptoms persisted. |
| **Karadas et al. (16)** | Turkey | 2015 | A 27-year-old male presented with numbness in his hands and legs, shortness of breath, and chest pain. His was tachycardic and had low oxygen saturation. Physical examination revealed bilateral rales. The Laboratory tests showed high CK-MB and troponin I. CXR displayed bilateral lower zones of infiltration. | Sinus tachycardia | LVEF=45% | SAV, steroid, H_1_ and H_2_ receptor antagonists, oxygen, IV fluid, diuretic and anticoagulant therapy, dopamine and dobutamine | Cardiac enzymes became negative on the 2^nd^ day of follow-up, and the patient was discharged. |
| **Khalilian et al. (17)** | Iran | 2021 | A 9-year-old girl, stung by Androctonus crassicauda, complained of pain at the sting site, shortness of breath, dizziness, cough, and profound sweating. On examination, she was tachycardic, hypotensive and tachypneic. Heart auscultation showed S_1_, S_2_, S_3_, and a systolic murmur. Crackles were heard in both lungs. CXR revealed an increased cardiothoracic ratio and increased pulmonary vascular markings. CK-MB and troponin I were raised. | Sinus tachycardia with diffuse ST-segment depression | Severe left ventricular  and also interventricular hypokinesia and reduced EF (25%) with mild mitral regurgitation | SAV, dopamine, ACE inhibitor (for reduction of preload and afterload) | The patient recovered completely. The left ventricle function returned to the normal level. |
| **Kir et al. (18)** | Turkey | 2011 | An 8-year-old boy presented with vomiting, cold, sweating, chills, and dyspnea. Examination showed cold and sweaty extremities, tachycardia, tachypnea, hypotension, and body temperature was 37.8°C. The laboratory tests revealed leukocytosis. Troponin and CK-MB were high. CXR displayed pulmonary edema. | Pathological ST depression in leads V_4_-V_6_. | Moderate left ventricular systolic dysfunction (EF=50%, FS=27%), mild left ventricular dilatation | SAV, dobutamine | Heart muscle enzymes decreased, ECG changes improved, and on the 10th day of admission, left ventricular function returned to normal on echocardiography. |
| **Lonati et al. (19)** | Italy | 2017 | A 25-year-old female, stung by a Leiurus quinquestriatus scorpion that presented with normal blood pressure, local acute pain, fever, tachycardia, dyspnea, and diffuse pulmonary rales. The laboratory tests showed an increase in troponin I and CPK-MB. CXR displayed severe pulmonary edema. | - | Significant LV systolic dysfunction with global hypokinesia and LVEF of 25% | Purified Polyvalent Anti-scorpion serum, prazosin, dobutamine, furosemide, hydrocortisone, levofloxacin, a proton pump inhibitor, acetaminophen, NSAID, clindamycin and enoxaparin. | Her clinical condition progressively improved with the normalization of myocardial necrosis indices and LVEF (67%) within nine days. Mild worsening of LVEF during the follow-up (53 to 60%) was seen. |
| **Maheshwari and Tanwar (20)** | India | 2012 | A 35-year-old female was stung by a red scorpion that presented with breathlessness, chest pain, and cold sweating. She had tachycardia, tachypnea, and blood pressure was 154/84 mmHg. There was an audible S_3_ with bilateral diffuse inspiratory basal crackles. Jugular venous pressure was raised. CK-MB and troponin I were elevated, CXR was compatible with pulmonary edema. | T-wave inversion in leads I, aVL, V_1_-V_2_, and ST segment elevation with concavity upward in leads II, III, aVF and V_5_- V_6_. | Hypokinesia of interventricular septum and inferior posterior wall, moderate mitral and tricuspid regurgitation, LVEF=28% | Antiplatelet drug and low-dose beta blockers. | Survived with the subsequent echocardiogram showing no regional wall motion abnormality, with well-preserved LVEF. |
| **Mahur et al. (21)** | India | 2014 | A 17-year-old female presented with shortness of breath, cough, and drowsiness. Blood pressure was 90/60 mmHg, and she had tachycardia and tachypnea. CXR revealed diffuse, fluffy, bilateral infiltrates. | Sinus tachycardia, left axis deviation, ST elevation of >1mm in all chest leads. | Global hypokinesia of left ventricle, severe left ventricle dysfunction (EF=25%), mild mitral and tricuspid regurgitation. | Oxygen with mask, ceftriaxone, hydrocortisone, dobutamine, furosemide, rabeprazole, digoxin. | She was discharged on the 6th day of admission without residual morbidity. |
| **Miranda and Maio (22)** | Brazil | 2014 | A 25-year-old woman developed several episodes of emesis, profuse sweating, mild respiratory distress, blood pressure of 220/110 mmHg and a heart rate of 156 bpm associated with sustained ventricular tachycardia. After admission, she developed hypotension, and poor peripheral perfusion, and worsening of respiratory distress. | sustained ventricular tachycardia with heart rate of approximately 300 bpm associated  with a right bundle branch block pattern, then sinus tachycardia and QTc prolongation | Echo on second day of admission: epression of the LVEF (30%) and severe hypokinesia, hyperkinetic apical region, on third day: LVEF: 41% and hypokinesia only in the basal segments | SAV, dobutamine, captopril, carvedilol, amiodarone (for sustained ventricular tachycardia) | Discharged and fully normalized echocardiography at 1-month follow-up |
| **Miranda et al. (23)** | Brazil | 2015 | A 7-year-old boy stung by Tityus serrulatus; presented with vomiting, profuse sweating, and respiratory distress, high blood pressure and heart rate. The laboratory data showed troponin I, NT-proBNP to be high. Bilateral fluffy shadows and a normal cardiac area compatible with pulmonary edema in CXR. CMR revealed an apical ballooning in the left ventricle associated with an LVEF of 29%, normal signal intensity of the basal myocardium but global edema of the midmyocardium and apical myocardium in T2. The late gadolinium images identified slight areas of diffuse necrosis/fibrosis | Sinus tachycardia associated with ST segment elevation in V_1_-V_3_, I, and aVL leads | Myocardial depression with a LVEF of 26% with important hypokinesia in the apical region | SAV, furosemide | On the sixth day, the patient was asymptomatic and discharged. The next echocardiography was entirely normal. Second CMR in 7 months was normal. |
| **Pradeep et al. (24)** | India | 2020 | A 19‑year‑old male presented with nausea, vomiting, profuse sweating, abdominal pain, and shortness of breath. He developed hypotension, tachycardia, and tachypnea. On examination, his extremities were cold, and he had cyanosis. Bilateral fine crackles in the lung fieldsand a loud S_3_ gallop at the apex of the heart were heard. The lab data showed elevated troponin I and CPK-MB. | Sinus tachycardia, right bundle branch block | Global left LV hypokinesia with moderate LV dysfunction (LVEF=40%) | Noradrenaline, dobutamine, furosemide | After seven days of hospital stay, he was discharged in a hemodynamically stable condition. His echocardiography was repeated near discharge, which showed normal LV function, LVEF=55%, and no pericardial effusion. |
| **Rahav and Weiss (25)** | Israel | 1990 | A 28-year-old man stung by a yellow scorpion developed sweating, tachycardia, and dyspnea. CXR showed white lungs. | Sinus tachycardia, ST-T change in inferolateral wall | Radionuclide ventriculography performed with a mobile gamma camera (MUGA scan) showed decreased contraction of the septum and the apical wall. LV contractility was reduced with EF of 27%. The right ventricle was enlarged with reduced function: EF=24% | Fluid therapy, SAV, morphine, furosemide, digoxin, steroid, continuous positive airway pressure mask with high oxygen flow | Survival with MUGA scan on discharge showing improved regional wall motion with LVEF of 60% and right ventricular EF of 41%. ECG changes resolved. Six months later there was still apical hypokinesis with LVEF of 56%. The right ventricle was still enlarged with an EF of 48%. |
| **Rahav and Weiss (25)** | Israel | 1990 | A 10-year-old boy, presented with irritability, priapism and hypersalivation. He was tachycardic but normotensive. The lungs were clear at first but then he developed dyspnea and CXR showed pulmonary edema. | Diffuse ST-T changes, especially in the anterolateral wall | Radionuelide ventriculography revealed reduced global LV function (EF=41%) with hypokinesis of the anteroseptal and apical walls | SAV, atropine, diazepam, oxygen, furosemide | Survival |
| **Ratnayake1 et al. (26)** | Sri Lanka | 2016 | A 34-year old man stung by Hottentotta tamulus, with high blood pressure and tachycardia. He developed dyspnea and chest tightness. Cardiac troponin was elevated. Coronary angiogram one month after the event was normal. | diffuse ST segment changes | Severe LV dysfunction (EF=33%), global hypokinesia | Prazosin, acetaminophen, lidocaine, ice packs, furosemide, oxygen | Survival with normal follow-up echocardiography |
| **Sahin et al. (27)** | Turkey | 2014 | A 38-year-old male presented with cold, sweating, nausea, vomiting, and shortness of breath after a yellow scorpion sting. Examination revealed cold extremities and laryngeal edema. His blood pressure was 90/60 mmHg, tachypnea, low Sao_2_, and tachycardia. The laboratory tests revealed leukocytosis, high troponin, AST, ALT, creatinine, D-dimer, and pro-BNP. CXR displayed widespread consolidated areas in both lungs. | Sinus tachycardia, no significant ST and T waves changes | Global hypokinesis, EF=33%, mild mitral regurgitation | Scorpion antivenom, adrenalin, antihistamine, steroids, bronchodilator, intubation and mechanical ventilation, prazosin, nitroglycerine, dobutamine, spironolactone, furosemide | The next echocardiography revealed normal wall movement with left ventricle EF=60%. |
| **Sari et al. (28)** | Turkey | 2015 | A 4-year-old girl, stung by a yellow scorpion, presented with pulmonary edema and respiratory distress. The extremities were cold and pale. She had tachycardia and very low blood pressure. The laboratory tests revealed elevated troponin I, pro BNP, and CK-MB. | Sinus tachycardia | Systolic and diastolic dysfunction with an EF of 45% | Scorpion antivenom, doxazosin, dobutamine, spironolactone, furosemide | The next echocardiography was normal, and the laboratory values returned to normal. The patient was discharged on the 6th day of hospitalization without any sequelae. |
| **Sezen et al. (29)** | Turkey | 2010 | A 12-year-old boy presented with tachycardia and hypotension and his condition worsened leading to confusion and respiratory distress and eventually to intubation. Examination revealed bilateral basal rales, and a loud S_3_ gallop at the apex and apical systolic murmur. CXR demonstrated bilateral fluffy shadows indicative of pulmonary edema and cardiomegaly. | Sinus tachycardia and nonspecific ST-T wave changes | Left ventricular dilatation, low ejection fraction (35%), third-degree mitral insufficiency, and extremely elevated pulmonary artery pressure (70 mm Hg) | Antivenom, dopamine, dobutamine, diuretics, and steroids | The patient was extubated within 36 hours. Repeat echocardiography revealed near-normal cardiac dimensions and pulmonary artery pressure. |
| **Yildizdas et al. (30)** | Turkey | 2008 | An 18-month-old girl presenting with fever, unconsciousness, respiratory distress, tachypnea, and tachycardia. Work up revealed leukocytosis, positive troponin and CK-MB, and pulmonary edema on CXR. | ST elevation | LVEF=39%  LVFS=20 | SAV, prazosin, dopamine, dobutamine, nitroprusside, epinephrine, ketamine, helmet-delivered non-invasive pressure support ventilation | Seven days after admission, control echocardiography was normal and inotropic drugs were stopped. Discharged 12 days after admission. |
| **Yildizdas et al. (30)** | Turkey | 2008 | A 7-year-old boy presenting with fever, convulsions, tachydyspnea, and tachycardia. Work up revealed leukocytosis, positive troponin and CK-MB, and pulmonary edema on CXR. | ST elevation | LVEF=42%  LVFS=23 | SAV, prazosin, dopamine, dobutamine, nitroprusside, epinephrine, ketamine, helmet-delivered non-invasive pressure support ventilation | Ten days after admission control echocardiography was normal and inotropic drugs were stopped. Discharged 12 days after admission. |
| **Yildizdas et al. (30)** | Turkey | 2008 | A 5-year-old girl presenting with fever, tachydyspnea and tachycardia. Work up revealed leukocytosis, positive troponin and CK-MB, and pulmonary edema on CXR. | ST elevation | LVEF=35%  LVFS=20 | SAV, prazosin, dopamine, dobutamine, nitroprusside, epinephrine, ketamine, helmet-delivered non-invasive pressure support ventilation | Nine days after admission, control echocardiography was normal. She was discharged 12 days after admission. |
| **Abbreviations: LV: left ventricle, EF: ejection fraction, CXR: chest x-ray, CMR: cardiac magnetic resonance, CK-MB: creatine kinase-myocardial band, MUGA scan: multigated acquisition mobile gamma camera** | | | | | | | |

**References**

1. Abroug F, Ouanes I, Maatouk M, Golli M, Ouanes-Besbes L. Inverted Takotsubo syndrome in Androctonus australis scorpion envenomation. Clin Toxicol. 2018;56(5):381-3.

2. Agrawal A, Kumar A, Consul S, Yadav A. Scorpion bite, a sting to the heart! Indian J Crit Care Med. 2015;19(4):233-6.

3. Aslan N, Yildizdas D, Horoz OO, Arslan D, Varan C, Erdem S, et al. Severe Troponin I Elevation and Myocardial Dysfunction in a Child with Scorpion Sting. J Pediatr Intensive Care. 2018;7(4):219-24.

4. Bayar N, Kucukseymen S, Yuksel IO, Arslan S. Rapidly improving acute myocarditis after a scorpion sting. Turk Kardiyol Dern Ars. 2013;41(7):629-+.

5. Ben Jemaa A, Bahloul M, Kallel H, Turki O, Dlela M, Bouaziz M. Inverted Takotsubo Syndrome due to Severe Scorpion Envenomation: Report of one Case. Med Trop Sante Int. 2021;1(1).

6. Bompelli N, Reddy CR, Deshpande A. Scorpion bite-induced unilateral pulmonary oedema. BMJ Case Rep. 2018;2018.

7. Brand A, Keren A, Kerem E, Reifen RM, Branski D. Myocardial damage after a scorpion sting: long-term echocardiographic follow-up. Pediatr Cardiol. 1988;9(1):59-61.

8. Bucaretchi F, De Capitani EM, Fernandes CB, Santos TM, Zamilute IAG, Hyslop S. Fatal ischemic stroke following Tityus serrulatus scorpion sting in a patient with essential thrombocythemia. Clin Toxicol. 2016;54(9):867-70.

9. D'sa SR, Peter JV, Chacko B, Pichamuthu K, Sathyendra S. Intra-aortic balloon pump (IABP) rescue therapy for refractory cardiogenic shock due to scorpion sting envenomation. Clin Toxicol. 2016;54(2):155-7.

10. Dias LS, Vivek G, Manthappa M, Acharya R. Delayed presentation of scorpion sting with cardiogenic shock. BMJ Case Rep. 2012;2012.

11. Dokur M, Dogan M, Yagmur EA. Scorpion-related cardiomyopathy and acute pulmonary edema in a child who is stung by Leiurus abdullahbayrami. Turk J Emerg Med. 2017;17(3):104-8.

12. Ersoy S, Yilmaz F, Sonmez BM, Kara AY, Guclu A. A Case of Acute Myocarditis and Rhabdomyolysis after a Scorpion Sting. J Emerg Med Case Rep. 2017;8(1):10-2.

13. Ismail M, Asaad N, Suwaidi JA, Kawari MA, Salam A. Acute myocarditis and pulmonary edema due to scorpion sting. Glob Cardiol Sci Pract. 2016;2016(1):e201610.

14. Izquierdo LM, Rodríguez Buitrago JR. Cardiovascular dysfunction and pulmonary edema secondary to severe envenoming by Tityus pachyurus sting. Case report. Toxicon. 2012;60(4):603-6.

15. Jain MK, Indurkar M, Kastwar V, Malviya S. Myocarditis and multiple cerebral and cerebellar infarction following scorpion sting. J Assoc Physicians India. 2006;54:491-2.

16. Karadas S, Gonullu H, Ebinc S, Kurt N, Golcuk Y. Pulmonary Edema and Myocarditis Developing Due to Scorpion Stings. J Clin Anal Med. 2015;6:98-100.

17. Khalilian MR, Tavallai Zavareh SA, Norouzi A, Ghazavi M, Goudarzi AA. Acute Myocarditis Due to Scorpion Sting in a 9-Year-Old Girl. CASE REPORTS IN CLINICAL PRACTICE. 2021;6(3):-.

18. Kir M, Karadas U, Yilmaz N, Saylam GS. Transient Myocarditis and Cardiomyopathy After Scorpion and Spider Envenomation. Guncel Pediatri. 2011;9(2):100-2.

19. Lonati D, Locatelli CA, Moro G, Catalano O. Cardiac magnetic resonance study of scorpion toxic myocarditis. QJM-An Int J Med. 2017;110(2):113-4.

20. Maheshwari M, Tanwar CP. Scorpion bite induced myocardial damage and pulmonary edema. Heart Views. 2012;13(1):16-8.

21. Mahur H, Jhirwal KS, Singh DP. Scorpion sting envenomation presenting with pulmonary oedema and subconjunctival haemorrhage. J Indian Acad Clin Med. 2016;17(4):316-8.

22. Miranda CH, Maio KT, Moreira HT, Moraes M, Custodio VIdC, Pazin-Filho A, et al. Sustained Ventricular Tachycardia and Cardiogenic Shock due to Scorpion Envenomation. Case Reports in Medicine. 2014;2014:251870.

23. Miranda CH, Braggion-Santos MF, Schmidt A, Pazin A. The first description of cardiac magnetic resonance findings in a severe scorpion envenomation Is it a stress-induced (Takotsubo) cardiomyopathy like? Am J Emerg Med. 2015;33(6):3.

24. Pradeep YKL, Bhogaraju VK, Pathania M, Rathaur VK, Kant R. Uncommon presentation of scorpion sting at teaching hospital. J Fam Med Prim Care. 2020;9(5):2562-5.

25. Rahav G, Weiss AT. Scorpion sting-induced pulmonary edema: scintigraphic evidence of cardiac dysfunction. Chest. 1990;97(6):1478-80.

26. Ratnayake RM, Kumanan T, Selvaratnam G. Acute myocardial injury after scorpion (Hottentotta tamulus) sting. Ceylon Med J. 2016;61(2):86-7.

27. Sahin C, Acar E, Beydilli H, Mert KU, Akin F, Altun I. Acute Toxic Myocarditis and Pulmonary Oedema Developing from Scorpion Sting. Int Cardiovasc Res J. 2015;9(1):55-9.

28. Sari F, Ceylan G, Sandal OS, Isguder R, Agin H. A case report: Myocarditis and pulmonary edema after scorpion sting. Izmir Dr Behcet Uz Cocuk Hastan Derg. 2015;5(3):209-13.

29. Sezen Y, Guntekin U, Buyukhatipoglu H, Kucukdurmaz Z. Rapidly improving, severe, acute myocarditis after a scorpion bite: an extremely rare complication and successful management. Am J Emerg Med. 2011;29(3):346-.

30. Yildizdas D, Yilmaz HL, Erdem S. Treatment of cardiogenic pulmonary oedema by helmet-delivered non-invasive pressure support ventilation in children with scorpion sting envenomation. Ann Acad Med Singap. 2008;37(3):230-4.
